# Customizing Ionic Micelles by Dynamic Coassembly of Sequence-Defined Peptoid Block Copolymers

**DOI:** 10.1021/acs.macromol.5c03586

**Published:** 2026-06-08

**Authors:** Erin Tsai, Meng Zhang, Guan-Rong Huang, Katelyn Hall, Richard E. Gillilan, Qingqiu Huang, Revati Kumar, Donghui Zhang

**Affiliations:** † Department of Chemistry and Macromolecular Studies Group, 5779 Louisiana State University, Baton Rouge, Louisiana 70803, United States; ‡ Department of Engineering and System Science, National Tsing Hua University, Hsinchu 30013, Taiwan; § Physics Division, National Center for Theoretical Sciences, Taipei 10617 Taiwan; ∥ MacCHESS (Macromolecular Diffraction Facility at CHESS), Cornell University, Ithaca, New York 14850, United States

## Abstract

Monomer sequence encodes the conformation and intra- and intermolecular interactions of sequence-defined polymers. Mixing different sequences represents an attractive strategy to modulate the intra- and interchain interactions to produce mesoscale assemblies with tunable size, geometry, and internal structures, provided that mixing different sequences is thermodynamically favored over self-sorting. In this study, we investigated the aqueous assembly of binary mixtures of sequence-defined peptoid block copolymers (BCPs) with discrete chain lengths and varying charge patterns, i.e., the charge number and relative positioning along the chains. Förster Resonance Energy Transfer (FRET) experiments revealed the dynamic coassembly of peptoid chains with varying charge patterns to produce hybrid micellar aggregates. Small-angle X-ray scattering (SAXS) analysis further showed that the size and aggregation number of the hybrid micelles can be controlled by adjusting the stoichiometry of distinct sequences in the solution. Moreover, the interfacial hydrophobicity of the micellar aggregates was tailorable by the molar ratio of distinct sequences, evidenced by the binding studies with 8-anilino-1-naphthalenesulfonic acid (ANS), whose fluorescence is sensitively dependent on the polarity of its environment. This study highlights the effectiveness of producing micellar assemblies with tunable structural characteristics and interfacial hydrophobicity by mixing sequence-defined peptoid chains with varying charge patterns. This strategy expands the accessible chemical design space from a limited sequence set, paving the way for high-throughput materials discovery of micellar assemblies for different applications, such as enhanced drug encapsulation and solubilization.

## Graphical abstract

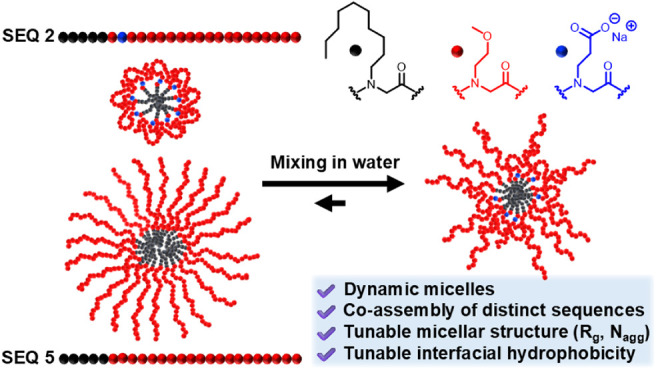

## Introduction

Coassembly of multiple components in an aqueous environment is a defining feature for constructing functional biomolecular complexes from biomacromolecules such as proteins and nucleic acids.^1–3^ Driven by the intricate interplay of various noncovalent interactions, such as electrostatic, hydrogen bonding, van der Waals, π–π stacking interactions, and solvation, biomacromolecules can form structurally complex assemblies.^4–6^ Notably, the precise spatial arrangement of charged residues has been shown to enable biomolecular recognition, self-assembly, and phase behavior. These processes are essential for normal cellular function and may lead to disease states if dysregulated.^7–10^ Additionally, biomacromolecular assemblies can change their charged state or the chemical identity of the charged residues to dynamically reorganize their structure in response to environmental changes. This adaptability allows for the formation of complex and functional assemblies from a limited set of building blocks.^11,12^

While the significance of charge patterns in governing biomolecular binding, assembly, and phase behavior is increasingly recognized, their application in synthetic systems remains less explored.^13–20^ Understanding how sequence-encoded electrostatic interactions modulate the coassembly of synthetic systems in solution is important for designing structural assemblies with programmable architectures and customizable functional properties. Advances in the synthesis of sequence-defined oligomers and polymers have facilitated precise control over nanoscale assemblies in water, where sequence-encoded intermolecular interactions play a decisive role in dictating the structural and dynamic outcomes.^13,15–24^ The coassembly of different sequences can further modulate the structure and stability of the molecular assemblies and introduce increased functionality in a spatially defined manner.^25–28^

The coassembly of surfactant mixtures has been investigated due to the potential for tailoring the stability of the coassembled structures and introducing additional chemical functionalities to the resulting solution aggregates.^29,30^ While surfactants tend to dynamically coassemble into aggregates with controlled morphology, they are limited in molecular architecture and chemical diversity relative to amphiphilic polymers. Consequently, there has been growing interest in the coassembly of amphiphilic polymers^28,31–36^ or synthetic peptides.^27,37,38^ Several studies on amphiphilic polymer mixtures have revealed a distinct outcome of coassembly or self-sorted assembly in solution, depending on the molecular architecture and chemical composition of the constituting polymers.^27,28,31–33,37,38^ For example, Imai et al. demonstrated that binary mixtures of facially amphiphilic random copolymers having identical hydrophilic PEG side chains and different hydrophobic side chains (butyl and dodecyl pendant groups) self-sorted into two discrete micelles instead of forming a coassembly in aqueous solution.^31^ Moreover, Timmers et al. have reported the formation of hybrid micelles formed from the coassembly of binary mixtures of precision polyurethane ionomers with linear amphiphilicity. Hydrophobic polyurethane can also coassemble with precision polyurethane ionomers, resulting in larger hybrid micelles.^28^ This study highlighted the significant role of electrostatic interactions and hydrophobicity in determining the coassembly behavior of precision polymers.

Polypeptoids (or peptoids) are peptidomimetic polymers in which the substituent group is attached directly to the nitrogen atom in the polyglycine backbone instead of the α-carbon as in polypeptides.^39,40^ *N*-substitution in peptoids has eliminated the extended backbone hydrogen bonding and stereogenic centers, which are hallmarks of polypeptides. Consequently, the secondary structure of peptoids is dictated by the molecular characteristics of their *N*-substituents rather than the hydrogen bonding that stabilizes β-sheets and α-helices in polypeptides.^41,42^ Sequence control of peptoids enables precise tuning of secondary interactions, including electrostatics, van der Waals, hydrogen bonding, and hydrophobic interactions, leading to large peptoid structure libraries (Figure 1).^41^ These attributes make peptoids effective model systems for probing how sequence-encoded noncovalent interactions influence their conformation, self-assembly structure, and dynamics in solution and at interfaces.^13,15,16,21,23,24,43–48^

**Figure 1:**
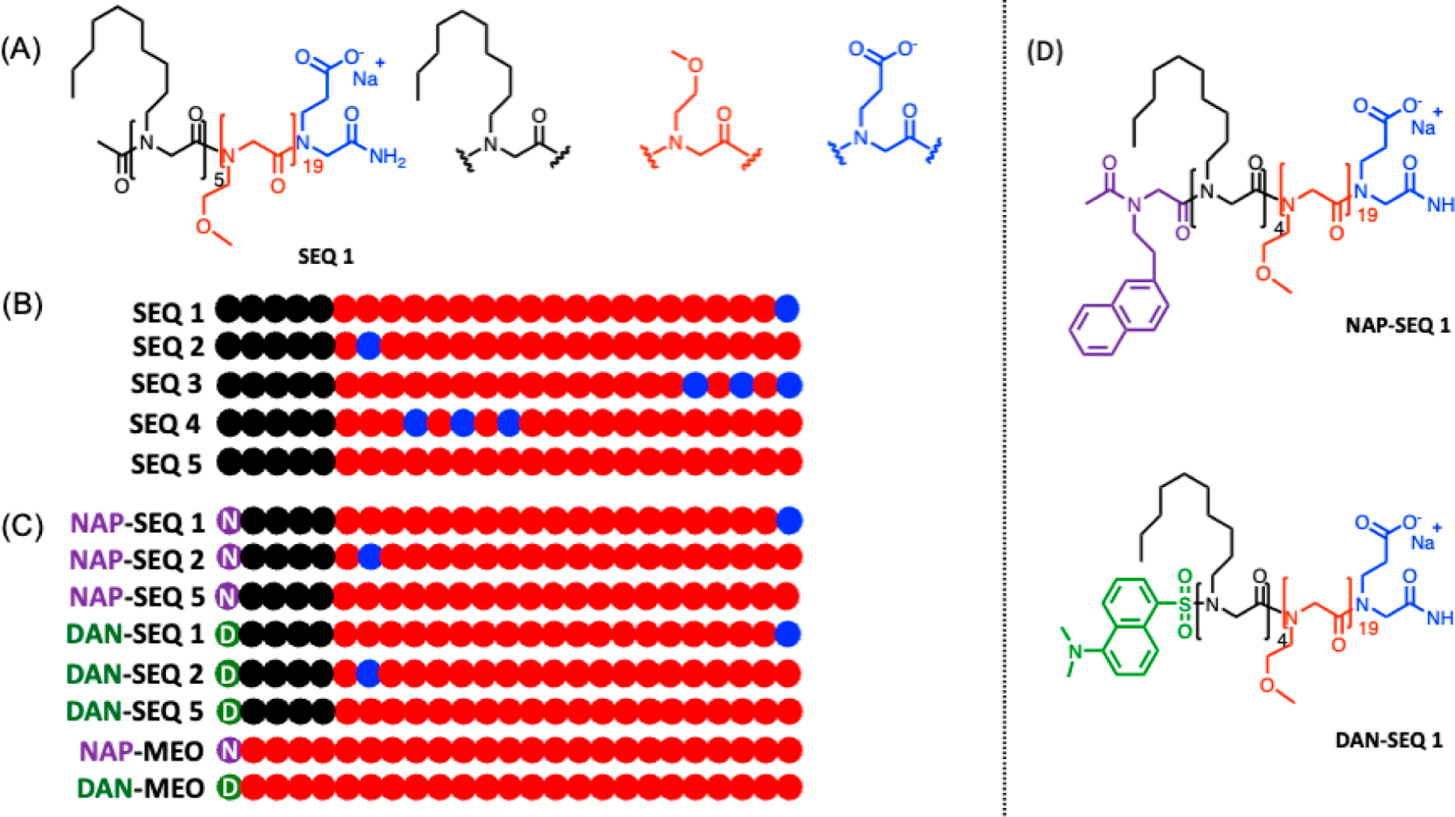
(A) Representative chemical structure of SEQ 1. (B) A sequence library of sequence-defined peptoid block copolymers (SEQ 1–5) comprised of *N*-decyl glycine (N_DC_, black), *N*-methoxyethyl glycine (N_ME_, red), and *N*-carboxyethyl glycine residues (N_CE_, blue). (C) The sequence library of sequence-defined peptoid block copolymers (SEQ 1, 2, 5) comprised of *N*-decyl glycine (black sphere), *N*-2-methoxyethyl glycine (red sphere), *N*-2-carboxyethyl glycine residues (blue sphere), *N*-acyl-*N*-2-naphthalenylethyl glycine (purple sphere), and *N*-dansyl group (light green sphere). (D) Representative chemical structures of the fluorophore-labeled peptoids, NAP-SEQ 1 and DAN-SEQ 1.

Previous studies on amphiphilic sequence-defined peptoid block copolymers (BCPs) have yielded important insights into the nanoscale and atomistic structures of their tunable micellar assemblies. The charge patterns of amphiphilic peptoid BCPs were shown to effectively modulate the micellar structures (aggregation number, micellar size, etc.) and intermicellar interaction potentials in dilute and semidilute aqueous solutions in a precisely controlled manner.^15,24,49^ MD simulations revealed that the charge patterns dictate the micellar structure by balancing the interplay of hydrophobic interactions, electrostatic repulsion, and solvation of charged monomers.^23^ The micellar assemblies of sequence-defined peptoid BCPs can undergo distinct pH-induced structural reorganization, which is modulated by the positioning of a single anionic monomer along the hydrophilic block.^43^ It has also been shown that the interfacial structure and water dynamics of the sequence-defined peptoid micellar interface can be tuned by altering the positions of the charged monomers along the chains.^16^

While prior studies on sequence-defined peptoid BCPs have demonstrated the importance of charge patterning in controlling micellar structure and pH-responsive behavior in aqueous solution,^15,43^ it remains unclear whether these micelles are dynamic assemblies capable of interchain exchange and coassembly or instead represent kinetically trapped structures. More broadly, in amphiphilic block copolymer systems, dynamic assemblies allow adaptive reorganization and broaden the design space by enabling structure and function to be tuned through chain mixing, whereas kinetically trapped assemblies are limited to properties established at formation. Resolving this distinction is, therefore, essential to determining whether sequence mixing can serve as a general strategy for accessing tunable hybrid micellar structures.

To address this gap, we investigated the aqueous coassembly of sequence-defined ionic peptoid block copolymers in aqueous solution. FRET studies revealed that the peptoid micelles are dynamic and undergo facile chain exchange. Coassembly of distinct peptoid sequences produces hybrid micelles with tunable size, aggregation number, and interfacial hydrophobicity, as controlled by sequence selection and stoichiometry, confirmed by small-angle X-ray scattering (SAXS) analyses and an environmentally sensitive dye-binding assay. Beyond offering fundamental insights into the sequence effects on ionic micellar assembly, this work presents a modular approach for diversifying the design space of tunable micellar assemblies, with potential for high-throughput fabrication and applications such as drug encapsulation and solubilization.

## Results and Discussion

### Synthesis and Characterization of Sequence-Defined Polypeptoid BCPs

A library of sequence-defined peptoid BCPs (SEQ 1–5) comprised of *N*-decyl glycine (black), *N*-2-methoxyethyl glycine (red), and *N*-2-carboxylethyl glycine residues (blue) were synthesized by the submonomer method (Figure 1) as previously reported.^15,24,50,51^ MALDI-TOF MS and HPLC analysis confirmed the successful synthesis of the peptoid BCPs with purity levels in the 86–96% range (Figure S1–S2, Table S1). Impurities were mainly deletion sequences missing one to five N_ME_ residues. SEQ 1 to SEQ 5 were previously shown to form spherical micelles in water (pH = 9.0), and the micellar size and internal structure (i.e., polymer and water distribution) can be precisely tuned by controlling the charge patterns of the peptoid BCPs.^15^ In addition, the ionic peptoid micelles (SEQ 1 and SEQ 2) can undergo structural reorganization with a notable change of aggregation number in response to solution pH changes, suggesting a dynamic characteristic of the micellar assemblies in solution.^43^ To investigate whether different peptoid sequences can coassemble in aqueous solution, we synthesized analogous sequence-defined peptoid BCPs with naphthalene (NAP) and 5-(*N*,*N*-dimethylamino)­naphthalene-1-sulfonyl (DAN) moieties, which are fluorophores commonly used in FRET experiments. As NAP and DAN moieties (Figure 1) are hydrophobic, with estimated Hanson parameters of δ_T_ = 23.9 MPa^1/2^ and δ_T_ = 24.1 MPa^1/^,^2^ respectively,^52–54^ labeling the peptoid BCP chains with these fluorophores may influence the thermodynamic propensity and dynamics of their assembly in aqueous solution. To minimize changes in the overall hydrophobicity of the labeled peptoid chains, we replaced the terminal *N*-decyl glycine residue (δ_T_ = 19.1 MPa^1/2^)^52–54^ with the fluorophore moieties (Figure 1, Table S2). The successful synthesis and purity of these fluorophore-labeled peptoid BCPs were confirmed by HPLC and MALDI-TOF MS analysis (Figure S1–S2, Table S1).

### Fluorescence Energy Transfer (FRET) Experiments

To investigate whether the peptoid micelles are dynamic assemblies capable of chain exchange and mixing, we began with neutral and single-charged sequences. If these micelles are indeed dynamic, peptoid sequences with higher ionic monomer content (e.g., triple-charged) are expected to form dynamic assemblies due to their enhanced water solubility. To start, micellar solutions of SEQ 1, SEQ 2, and SEQ 5 peptoid sequences (3.0 mg/mL, pH 9.0) were individually prepared with 10 wt % of chains consisting of fluorophore-tagged sequences, i.e., NAP-SEQ 1, NAP-SEQ 2, NAP-SEQ 5, DAN-SEQ 1, DAN-SEQ 2, and DAN-SEQ 5, respectively (details in Supporting Information).^24,55^ The pH was adjusted to 9.0 to ensure complete ionization of the carboxylic acid groups on the polymers containing ionizable groups, given a typical p*K*_a_ of 5–6 for these peptoid sequences.^15,24^ The peptoid solutions containing 10 wt % of NAP- or DAN-labeled sequences and 90 wt % unlabeled counterparts were heated at 80 °C for 3 h to promote micellar equilibration before mixing the two micellar solutions with NAP-labeled sequences or DAN-labeled sequences at room temperature overnight (≥8 h). Our previous studies have shown that thermal annealing of the peptoid micellar solutions effectively eliminated the formation of sporadic, large micellar aggregates observed in nonannealed conditions, yielding spherical micelles with narrower size distributions in solution.^24,43,55^ Two peptoid micellar solutions, each labeled with NAP or DAN fluorophores, were then mixed at an equal molar ratio of two different peptoid sequences (final total peptoid concentration = 3.0 mg/mL) and allowed to equilibrate for (≥6 h) prior to FRET measurements.

It should be noted that fluorescently labeled peptoid sequences were incorporated at a low fraction (10.0 wt %) relative to the unlabeled counterparts to form micelles in the FRET experiments. Dynamic light scattering analysis of a micellar solution containing 5 wt % DAN-SEQ 5, 5 wt % NAP-SEQ 5, and 90 wt % unlabeled SEQ 5 exhibited a similar size distribution to that consisting entirely of unlabeled SEQ 5 (Figure S3), indicating that the incorporation of a low fraction of fluorophore-labeled sequences does not significantly perturb the micellization and the resulting micellar structure.

The fluorescence emission profiles for the micellar solutions containing two different peptoid sequences (SEQ 1 and SEQ 5, SEQ 2 and SEQ 5, SEQ 1 and SEQ 2) revealed two distinct fluorescent emission maxima at 350 and 530 nm, attributed to the NAP (donor) and DAN (acceptor) labeled peptoids, respectively (Figure 2A–C). Importantly, the emission intensity at 530 nm for the micellar solution containing two different peptoid sequences is significantly increased relative to that of the micellar solution containing only the acceptor (DAN)-labeled peptoids. This is accompanied by a noticeable reduction of the emission intensity at 350 nm relative to that of the micellar solution containing only the donor (NAP)-labeled peptoids. These results clearly indicate the occurrence of FRET between DAN-peptoid and NAP-peptoid chains by mixing the two single-sequence peptoid micellar solutions. To further assess the efficiency of the fluorescence resonance energy transfer process, we calculated the FRET efficiency (*E*) from the fluorescence emission profiles using the following equation (eq 1):^56^

1 $$E=1-I_{\mathrm{NAP}}/I_{\mathrm{NAP},0}$$

where *I*_NAP_ and *I*_NAP,0_ and stand for the steady-state fluorescence intensity of the donor with or without the presence of the acceptor, respectively. The FRET efficiency obtained by mixing two peptoid micellar solutions (SEQ 1:SEQ 5, SEQ 2:SEQ 5, and SEQ 1:SEQ 2) was found to be in the 56–71% range (Figure 2D). In addition, the corrected acceptor-to-donor ratio (*I*_corr,DAN_ */I*_NAP_) (Figure S4) accounting for both the changes of the donor and acceptor emission intensities has been calculated using eq S6 and found to be in the 0.16–0.66 range, consistent with the observed trend in *E*.^31,57^ While the corrected acceptor-to-donor ratio of 0.16 was low for the SEQ 2 and SEQ 5 mixture, this value does not correspond to absolute FRET efficiency. Not all quenched donor energy is recovered as acceptor fluorescence due to nonradiative decay, potential acceptor quenching, and differences in the local dielectric environment from aggregation.^31,57^

**Figure 2:**
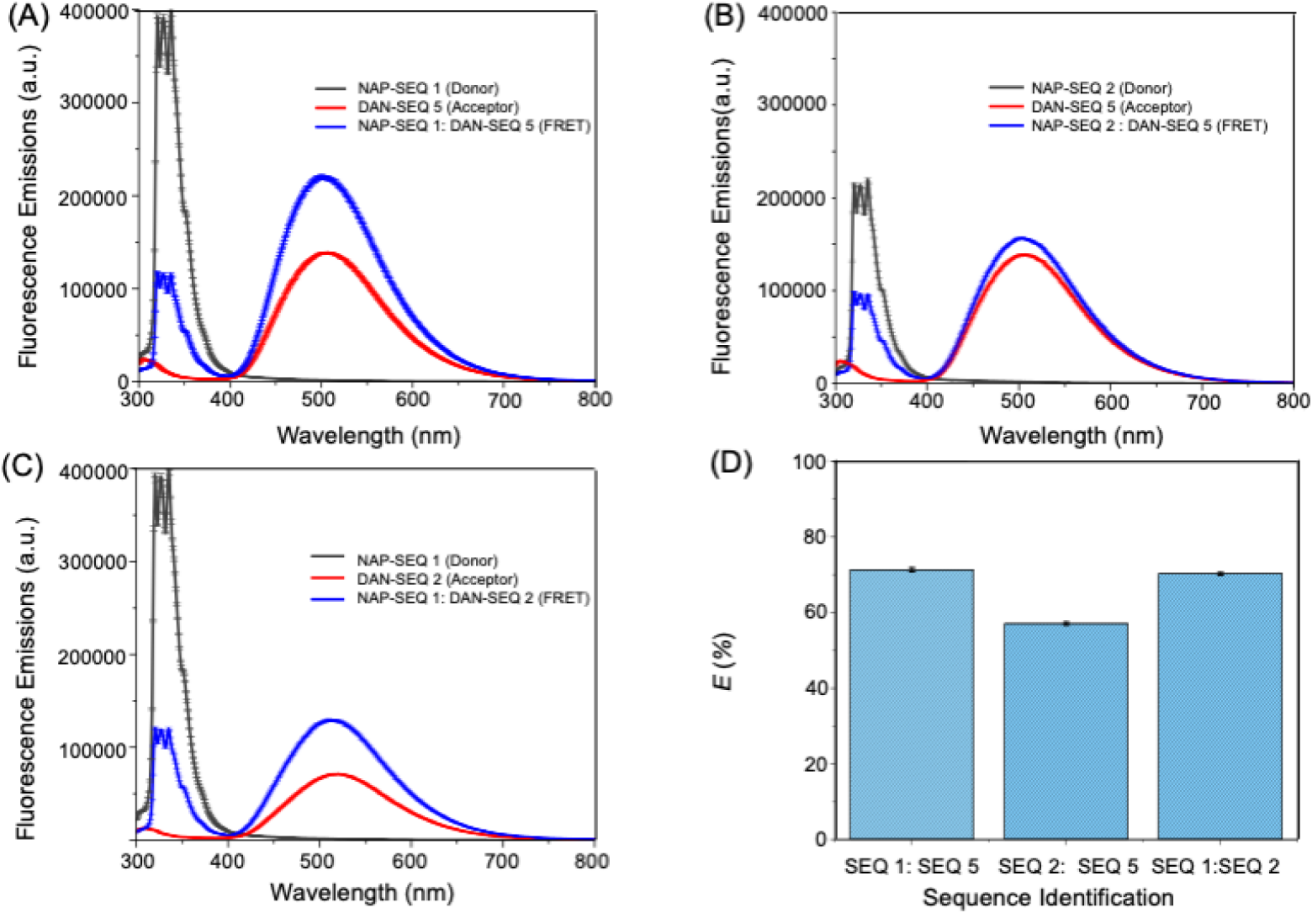
Fluorescence emission spectra of micellar solutions containing two different sequence-defined peptoid BCPs in a 1:1 molar ratio: (A) SEQ 1 and SEQ 5, (B) SEQ 2 and SEQ 5, (C) SEQ 1 and SEQ 2, and the respective single-sequence peptoid micellar solution, as well as (D) the corresponding observed FRET efficiency (*E*).

The FRET efficiency (*E*) is related to the Förster radius (*R*_0_) of the donor and acceptor fluorophore and their separation distance­(*r*) in solution, as described by the following equation (eq 2):^57–59^

2 $$E=\frac{1}{1+{\left(\frac{r}{R_{0}}\right)}^{6}}$$

The Förster radius (*R*_0_) of the donor and acceptor fluorophore is determined by the molecular characteristics of the fluorophores, orientation, and their environment, as described by the following equation (eq 3):^57–59^

$$R_{0}=0.2108{[\frac{\kappa^{2}\Phi_{0}J}{n^{4}}]}^{1/6}$$ 3

where κ is the orientation factor (*κ* ^2^ = 2/3 in solution), Φ_0_ is the quantum yield of the donor, *J* is the degree of spectral overlap for the donor and acceptor emissions, and *n* is the refractive index of the medium.

The Förster radius (*R*_0_) for the NAP and DAN fluorophores is 23.3 ± 0.4 Å in water,^59^ which is much smaller than the radius of gyration of the peptoid micelles (*R*_g_ = 43.7 ± 0.5 Å–56.8 ± 0.5 Å) used in this study (Figure 4). In addition, the FRET fluorophore is attached to the hydrophobic block end of the amphiphilic peptoid chains. If single-sequence peptoid micelles are kinetically trapped and unable to undergo chain exchange or mixing, the FRET donor and acceptor fluorophores inside separate micelles would remain separated by distances greater than the Förster radius. In this case, there would be minimal FRET (*E* = 0.47–2.2%, estimated using the above *R*_g_, *R*_0_, and eq 2). By contrast, if the micelles are dynamic assemblies, donor and acceptor fluorophores could colocalize within the same micelle interior through chain exchange and mixing, resulting in significant FRET. As the experimental FRET efficiency (*E*) is significantly higher in the 56–71% range (Figure 2D), this strongly indicates the colocalization of the NAP-peptoid and DAN-peptoid chains within the same micelles, supporting the formation of hybrid micelles by dynamic coassembly of the different peptoid sequences.

The FRET results clearly indicate that these peptoid micelles are dynamic assemblies capable of chain exchange and mixing between micelles, rather than kinetically frozen structures incapable of chain exchange (Figure 3). In addition to micelles, there is also a low concentration (critical micelle concentration level) of peptoid unimers in solution. To verify whether the hydrophobic fluorophores (NAP and DAN) may induce the association of peptoid unimers, thus contributing to the observed FRET in the above study, we synthesized two control sequences of fluorescently labeled peptoids consisting entirely of hydrophilic *N*-2-methoxyethyl glycine residues (i.e., NAP-MEO and DAN-MEO) and conducted fluorescent measurements of the solution containing these two sequences in equal molar ratio over a total peptoid concentration range of 0.0625–0.75 mg/mL (Figure S5). Note that this concentration range covers the fluorophore-labeled peptoid concentration used in the earlier FRET experiments (0.375 mg/mL) (Figure 2). Fluorescent measurements revealed the lack of FRET between the two sequences in these concentration ranges (Figure S5–S6), suggesting that the hydrophobic fluorophores do not induce significant association of peptoid unimers. Thus, the observed FRET by mixing different sequence-defined peptoid micellar solutions (SEQ 1 and SEQ 5, SEQ 2 and SEQ 5, and SEQ 1 and SEQ 2) can be solely attributed to the colocalization of fluorophore-labeled peptoid chains in the hybrid micelles resulting from dynamic micellar mixing (Figure 2).

**Figure 3:**
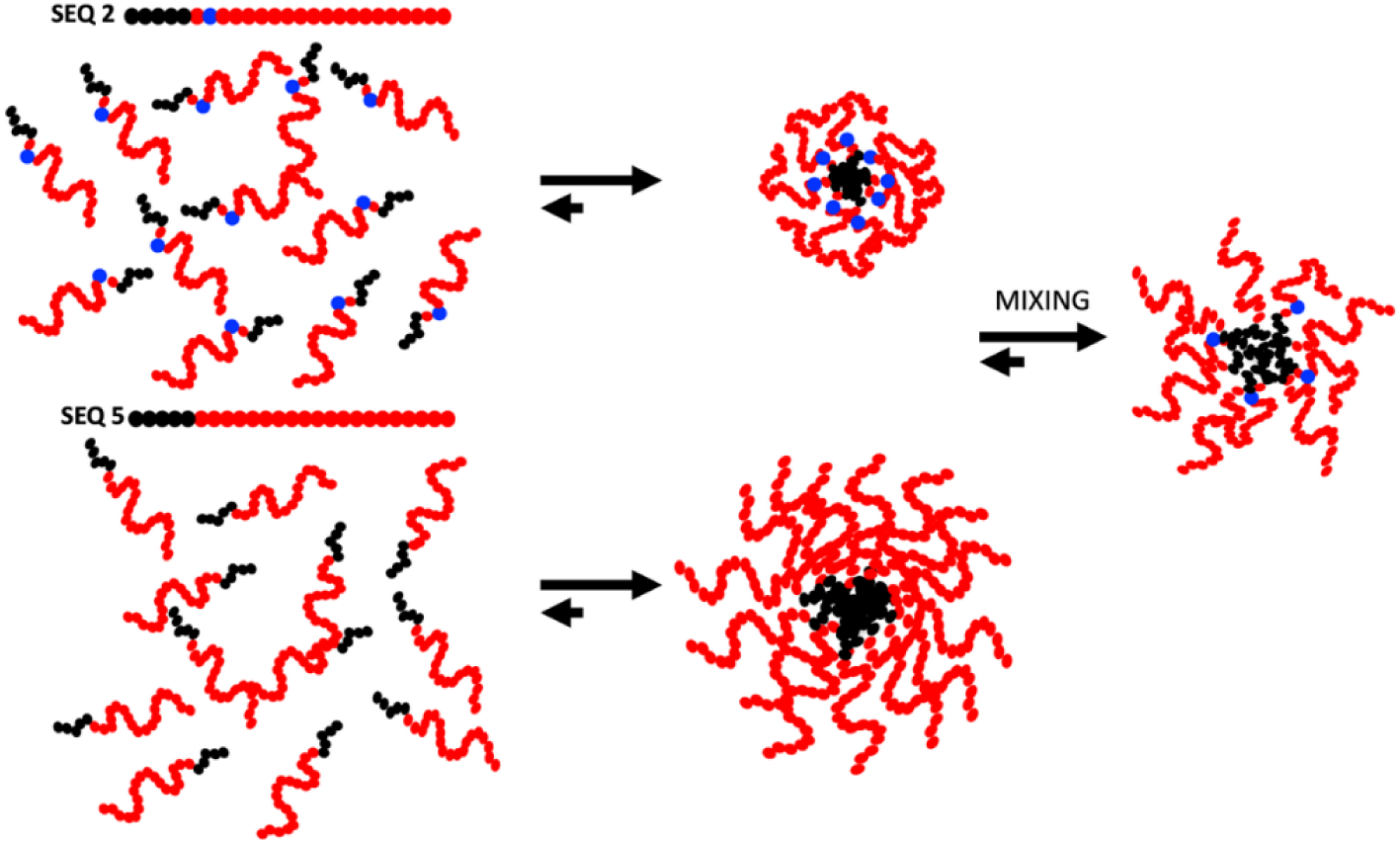
Schematic illustration of the proposed dynamic unimer exchange between two single-sequence peptoid micelles, resulting in the formation of hybrid micelles consisting of both SEQ 2 and SEQ 5 peptoid chains. In solution, single-sequence micelles of SEQ 2 and SEQ 5 (top and bottom left) exist in dynamic equilibrium with their respective unimers. If sequence mixing is thermodynamically favored, hybrid micelles consisting of both SEQ 2 and SEQ 5 can form by dynamic chain exchange. Color spheres represent *N*-decyl glycine (black), *N*-methoxyethyl glycine (red), and *N*-carboxyethyl glycine (blue) residues in the peptoid chains, respectively.

The trends in FRET efficiency (*E*) and the corrected acceptor-to-donor ratio (*I*_corr,DAN_ */I*_NAP_) varied among the different peptoid sequence mixtures (Figure 2D and Figure S4), indicating differences in the local dielectric environment and fluorophore orientation within the hybrid micelles.^57,59,60^ In particular, the local microenvironment can differ substantially between ionic and neutral peptoid micelles. Peptoid micelles containing ionic coronas (SEQ 1 and SEQ 2) are more hydrated and polar and, therefore, may result in a higher dielectric environment, whereas the neutral micelles of SEQ 5 may provide a more hydrophobic and compact local environment that affects fluorophore partitioning and emission behavior. These differences in the dielectric properties of the microenvironment and fluorophore partitioning within the micelles can influence donor–acceptor distance, orientation, and quantum yield, resulting in varying FRET efficiency.^57,59^

### Critical Micellar Concentration (CMC) and SAXS Measurements of Hybrid Micelles Consisting of Binary Mixtures of Sequence-Defined Peptoid BCPs

We prepared a series of hybrid micellar solutions of two different sequence-defined peptoid BCPs (SEQ 1: SEQ 5, SEQ 2: SEQ 5, and SEQ 1: SEQ 2) in controlled molar ratios (2:8, 4:6, 6:4, and 8:2) with a constant ionic strength ([NaCl] = 60 mM) and varying total peptoid concentrations (details in Supporting Information). Static light scattering (SLS) measurements were conducted to determine the critical micellar concentration (CMC) of the respective hybrid peptoid micelles.^61–63^ The absolute count rate at a backscattering angle of 175° was monitored at increasing concentrations of peptoid solutions, revealing a distinct transition point corresponding to the onset of micellization (Figure S7 and Table S3). The CMC of hybrid peptoid micelles (SEQ 1: SEQ 5, SEQ 2: SEQ 5, and SEQ 1: SEQ 2) and their respective single-sequence peptoid micelles (SEQ 1, SEQ 2, and SEQ 5) were found to be in the range of 0.163 ± 0.002–0.234 ± 0.008 mg/mL (Table S3), which is higher than previously reported values (0.02 ± 0.01–0.05 ± 0.02 mg/mL) for the respective single-sequence peptoid micelles.^24^ This can be attributed to the higher ionic strength ([NaCl] = 60 mM) in the peptoid micellar solutions used in the current study relative to those in the early study where no salt was added, which can alter the thermodynamics of micelle formation and the apparent CMC.

Small-angle X-ray scattering (SAXS) experiments were conducted on these hybrid micellar solutions (SEQ 1: SEQ 5, SEQ 2: SEQ 5, and SEQ 1: SEQ 2) with varying molar ratios of peptoid sequences (2:8, 4:6, 6:4, and 8:2) at 3.0 mg/mL total peptoid concentration (20 °C, pH = 9.0, [NaCl] = 60 mM) to gain information regarding the hybrid micellar structures in aqueous solutions. The SAXS profiles of all peptoid micellar solutions (Figure 4A,C,G) exhibited features indicative of a core–shell structure, evidenced by a near-plateau region at 0.0089 < *Q* < 0.02, followed by a gradual decrease of intensity and a clear minimum with increasing *Q* in the 0.02 < *Q* < 0.1 range. At the high *Q* range (0.12 < *Q* < 0.18), the scattering intensity exhibited a power law dependence on *Q* (i.e., *I*(*Q*) ∼ *Q* ^α^) with an exponent (α) in the −1.45 ± 0.02 to −1.90 ± 0.01 range, indicating swollen coil to more extended chain conformations of the micellar corona chains.^64^ For all peptoid micelles, the low *Q* scattering intensity exhibited a weak power law dependence on *Q* with an exponent in the −0.27 to −0.44 range (0.0089 < *Q* < 0.023 Å^–1^), consistent with the formation of zero-dimensional nanostructures. Thus, the Guinier approximation of the SAXS data (using criteria *R*_g_ × *Q* < 1.0) (Figure S8) was applied to determine the micellar aggregation number (*N*_agg_) and radius of gyration (*R*_g_) (Figure 4G,H, Table S3–S4) of all hybrid and single-sequence peptoid micelles.

**Figure 4:**
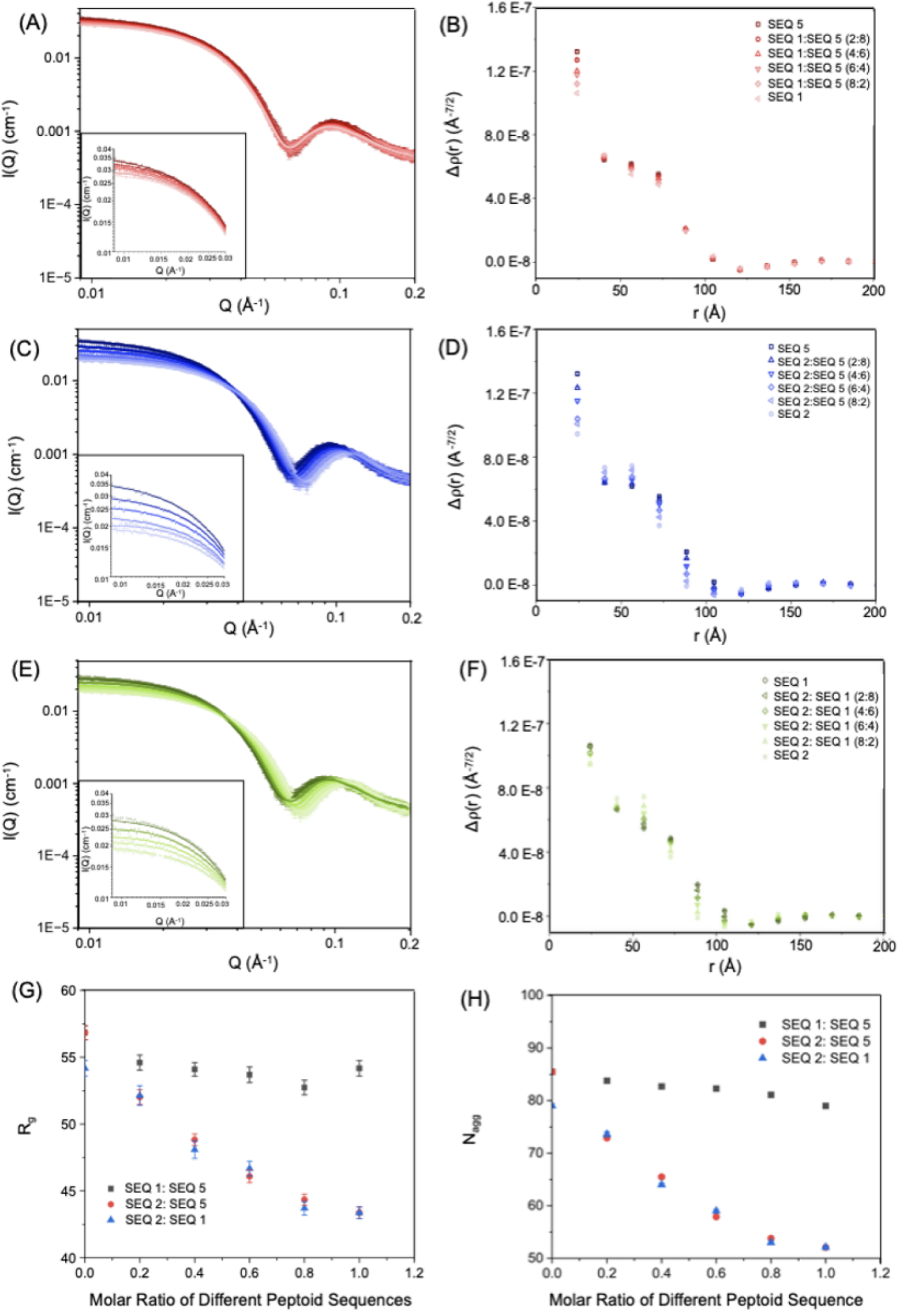
SAXS characterization revealing tunable hybrid micellar structures by mixing different sequences of peptoid BCPs. (A, C, E) SAXS data and the corresponding form factors obtained by the PhaseLift method (solid lines) of hybrid micelles consisting of two different sequence-defined peptoid BCPs in varying molar ratios ([peptoid] = 3.0 mg/mL, [NaCl] = 60 mM, pH = 9.0, 20 °C). (B, D, F) excess scattering length density profiles Δρ­(*r*) extracted by the PhaseLift method for various hybrid micelles. (A, B) SEQ 1: SEQ 5; (C, D) SEQ 2: SEQ 5; (E, F) SEQ 2: SEQ 1. (G) Plot of micellar size (*R*_g_) and (F) aggregation number (*N*_agg_) at varying molar ratios of two peptoid sequences (SEQ 1: SEQ 5, SEQ 2: SEQ 5, SEQ 2: SEQ 1). Error bars shown in (B, D, F) represent the uncertainty from the Δρ*(r)* reconstruction using the PhaseLift method and are smaller than the symbol size at the plotted scale.

Guinier plot analysis revealed the largest micellar aggregation number and radius of gyration (*N*_agg_ = 85.5 ± 0.5, *R*_g_ = 56.8 ± 0.5 Å) for the neutral micelle SEQ 5, followed by the ionic micelles SEQ 1 (*N*_agg_= 79.0 ± 0.4, *R*_g_= 54.2 ± 0.5 Å) and SEQ 2 (*N*_agg_= 52.1 ± 0.2, *R*_g_= 43.4 ± 0.4 Å), the trend of which is consistent with earlier reports.^15,24^ The ionic micelles (SEQ 1 and SEQ 2) exhibit a reduced degree of chain packing relative to the neutral micelles (SEQ 5) due to enhanced electrostatic repulsion in the former. The electrostatic effect is enhanced in the SEQ 2 micelle relative to the SEQ 1 micelle due to the positioning of the charged monomer at the hydrophilic block terminus in the latter versus at the hydrophobic/hydrophilic block junction in the former. Mixing different peptoid sequences has led to varying degrees of tunability in the resulting hybrid micellar structures. For example, hybrid micelles composed of SEQ 1 and SEQ 5 exhibited comparable micellar dimensions (*R*_g_) and aggregation numbers (*N*_agg_) in different molar ratios of the two peptoid sequences (Figure 4G,H, Table S3). By contrast, hybrid micelles composed of SEQ 2:SEQ 5 exhibit a systematic and notable decrease of *R*_g_ and *N*_agg_ with an increasing proportion of SEQ 2 in the sequence mixture (Figure 4G,H, Table S3). The same trend was also observed for the SEQ 2 and SEQ 1 hybrid micelles as the ratio of SEQ 2 to SEQ 1 increased (Figure 4G,H, Table S3). These results are consistent with the augmented electrostatic effect on micelle formation resulting from repositioning the ionic monomer closer to the hydrophobic/hydrophilic junction (SEQ 2) relative to the hydrophilic terminus (SEQ 1).

To obtain a real-space description of intramicellar structure, the excess scattering length density profile, Δρ*(r)*, was reconstructed from the SAXS data using a model-free PhaseLift method without assuming a particular particle geometry.^49,65^ The Δρ*(r)* profile (Figure 4B,D,F) provides a radially averaged measure of the micellar internal density distribution, capturing the intramicellar distribution of polymer segments and invasive water.^49,65^ All micelles exhibited a core–shell structure, as shown by Δρ­(*r*) profiles with an initial gradual decay (*r* = ∼30–70 Å) followed by a sharper decline toward zero (Figure 4B,D,F). Δρ*(r)* profiles of SEQ 1/SEQ 5 hybrid micelles revealed a gradual decrease in core compaction, while their overall micellar spatial extension remained comparable as the ionic sequence content (SEQ 1) increased. In contrast, SEQ 2/SEQ 5 hybrid micelles exhibited a more pronounced reduction in core compaction and spatial extension with increasing ionic sequence content (SEQ 2). A similar trend was observed for SEQ 2/SEQ 1 hybrid micelles, in which both parameters decreased as the SEQ 2:SEQ 1 ratio increased. The micellar boundary radius (*R*_m_), which characterizes the micellar spatial extension, was determined by interpolating the point at which Δρ­(*r*) decays to zero. The trend in micellar spatial extension (*R*_m_) (Figure S9) is consistent with trends in the micellar dimension (*R*_*g*_) and aggregation number (*N*_agg_) determined by Guinier analysis (Figure 4). These findings demonstrate that hybrid micelles with controllable size and degree of chain compaction can be obtained by adjusting the peptoid sequence composition and molar ratio of the two sequences.

### Probing the Interfacial Hydrophobicity of Hybrid Peptoid Micelles Using an Environmentally Sensitive Chromophore

We conducted fluorescent measurements of single-sequence peptoid micelles and hybrid micelles consisting of two different peptoid sequences using 8-anilinonaphthalene-1-sulfonic acid (ANS) to characterize the interfacial hydrophobicity differences of these micelles. ANS is an environmentally sensitive chromophore exhibiting significant enhancement of fluorescence emission corresponding with a red-shift in hydrophobic environments relative to polar ones and has been used for characterizing internal and external hydrophobic binding sites of proteins.^66–68^ All micelles with ANS dyes exhibited a fluorescence maximum at *c.a.* 500 nm. Among all the single-sequence peptoid micelles (SEQ 1–SEQ 5, Figure 1), the neutral SEQ 5 micelles exhibited the highest fluorescence emission at 500 nm, while the inner-charged SEQ 4 with three ionizable monomers has the least (Figure 5). This aligns with previous findings that neutral SEQ 5 micelles have the largest micellar size and aggregation number relative to other peptoid micelles based on SAXS analysis (Figure 4).^15^ The neutral micelles have more compact chain packing and reduced water penetration into the micellar interior, resulting in a more hydrophobic micellar interface, relative to the ionic micelles. To simplify the comparison of interfacial hydrophobicity of different ionic micelles, all fluorescence emission data using ANS dye were normalized against the emission maximum of SEQ 5, which gives the most intense fluorescence emission.

**Figure 5:**
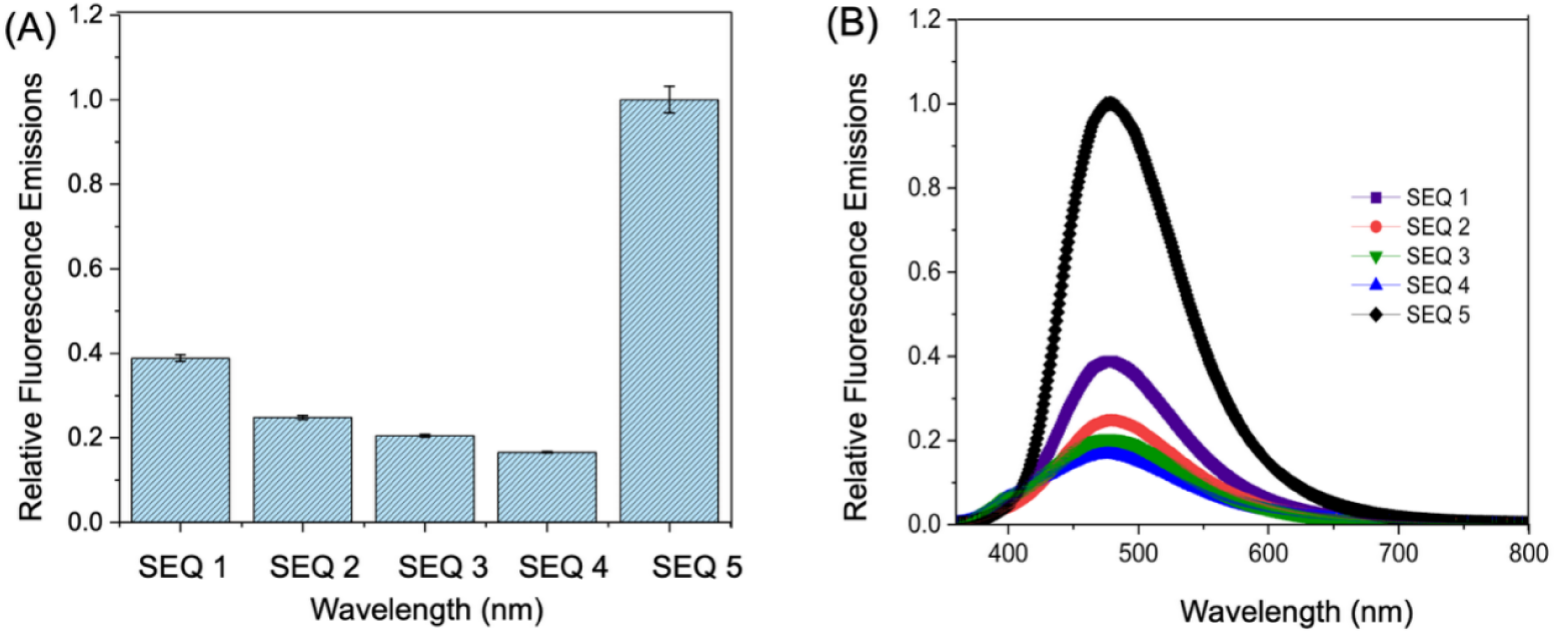
(A) The relative maximum fluorescence emissions of the single-sequence peptoid micelles SEQ 1–SEQ 5 and (B) their corresponding fluorescence emission spectra of pure, self-assembled micelles. All spectra show an intensity maximum at 480–500 nm. Error bars are the standard deviation from triplicate measurements.

The terminally charged SEQ 1 micelles exhibited distinct intensity in the fluorescence emission profile as compared to the inner-charged SEQ 2 micelles, suggesting that the ionic monomer’s position relative to the hydrophobic/hydrophilic junction influences micellar packing and interfacial hydrophobicity (Figure 5). The SEQ 1 micelles exhibit a higher fluorescence intensity than the SEQ 2 micelles, which aligns with SEQ 1’s larger micellar *R*_g_ and *N*_agg_ expected of more compact micelles (Figure 4). SEQ 3 and SEQ 4 micelles consisting of triply charged peptoid sequences each exhibited weaker fluorescence emission than their singly charged counterparts (Figure 5), consistent with the formation of less compact micelles by the triply charged sequences to alleviate the otherwise greater electrostatic repulsions.^15^ Similar to the peptoid micelles consisting of singly charged sequences (SEQ 1, SEQ 2), the micelles of triply charged sequences (SEQ 3, SEQ 4) also exhibited decreased interfacial hydrophobicity when the ionic monomers are placed closer toward the hydrophobic block junction (Figure 5).

Fluorescence measurements have revealed that the interfacial hydrophobicity of the hybrid micellar assemblies can be systematically tuned by adjusting the molar ratio of a singly charged sequence (SEQ 1 or SEQ 2) to a neutral sequence (SEQ 5) in solution (Figure 6A). Increasing the fraction of the singly charged sequence (SEQ 1:SEQ 5, SEQ 2:SEQ 5) was found to correlate with a decrease in interfacial hydrophobicity, attributable to the increased charge content within the micelles, as evidenced by a systematic reduction in fluorescence emission at 500 nm (Figure 6A). Additionally, hybrid micelles composed of SEQ 2 and SEQ 5 exhibited a wider tunable range of interfacial hydrophobicity relative to the hybrid micelles based on SEQ 1 and SEQ 5 (Figure 6B). This is consistent with the placement of a single ionizable monomer at the hydrophobic/hydrophilic block junction in SEQ 2, which more effectively promotes water penetration into the micellar interface.^24^

**Figure 6:**
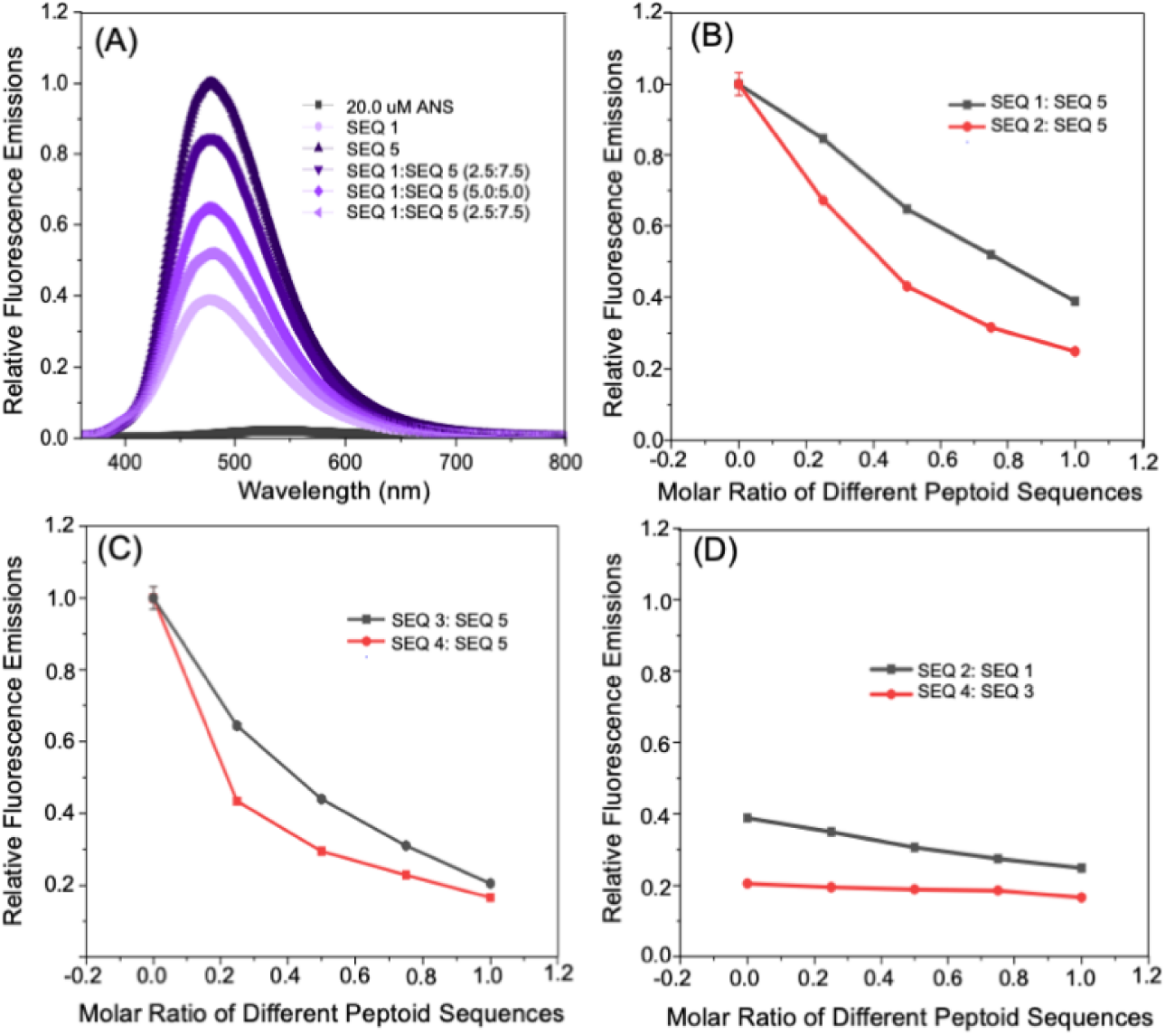
(A) Representative fluorescence emission spectra of hybrid peptoid micelles consisting of a binary mixture of a singly charged sequence (SEQ 1) and a neutral sequence (SEQ 5) in varying molar ratios. Plots of relative maximum fluorescence intensity of hybrid micelles at different molar ratios of the two different peptoid sequences: (B) a singly charged sequence and a neutral sequence (SEQ 1: SEQ 5, SEQ 2: SEQ 5); (C) a triply charged sequence and a neutral sequence (SEQ 4: SEQ 5, SEQ 3: SEQ 5); (D) two singly charged sequences (SEQ 2: SEQ 1) and two triply charged sequences (SEQ 4: SEQ 3). The spectra show an intensity maximum at 480–500 nm. Error bars are the standard deviation from triplicate measurements.

Similarly, mixing a triply charged peptoid sequence (SEQ 3 or SEQ 4) with a neutral sequence (SEQ 5) at varying molar ratios also afforded hybrid micelles with tunable interfacial hydrophobicity (Figure 6C). The maximum fluorescence intensity of the hybrid micelles containing the triply charged sequences (SEQ 3 or SEQ 4) was lower than that of hybrid micelles having the singly charged sequences (SEQ 1 or SEQ 2), consistent with the reduced interfacial hydrophobicity of the former micelles arising from the higher charge content (Figure S10A-B, Table S5). Meanwhile, the hybrid micelles containing the triply charged sequences (SEQ 3 or SEQ 4) exhibited a broader tunable range of interfacial hydrophobicity relative to the hybrid micelles incorporating the singly charged sequences (SEQ 1 or SEQ 2) (Figure S10A-B and Table S5).

While micellar interfacial hydrophobicity can also be adjusted by systematically varying the molar ratio of two singly charged peptoid sequences (SEQ 2 to SEQ 1) (Figure 6D), the tunable range is notably narrower than that of the hybrid micelles composed of one singly charged (SEQ 1 or SEQ 2) and one neutral sequence (SEQ 5) (Figure 6D). By contrast, mixing two triply charged sequences (SEQs 3 and 4) in varying molar ratios produced no significant change in the interfacial hydrophobicity, reflecting the nearly identical interfacial hydrophobicity of the corresponding single-sequence micelle (Figure 6D). Collectively, these results indicate that mixing charged and neutral sequences is a particularly effective strategy for tailoring the interfacial hydrophobicity of the peptoid micelles.

## Conclusions

We have demonstrated that binary mixtures of sequence-defined peptoid block copolymers self-assemble in aqueous solution into dynamic micelles that undergo facile chain exchange and mixing, leading to the formation of hybrid micelles. By varying peptoid sequences and stoichiometry, key micellar properties, including micellar size, aggregation number, and interfacial hydrophobicity, can be systematically tailored. These findings highlight sequence-encoded electrostatic interactions as powerful design parameters for programming ionic micellar assemblies. This work has established charge-patterned peptoid polymers as a versatile platform for engineering micellar assemblies with programmable architecture and interfacial environments and presented an approach that is potentially well-suited for high-throughput materials discovery and holds promise for a wide range of applications, including drug encapsulation and solubilization, water purification, and chemical catalysis.

## Supplementary Material



## Notes

Synthetic procedures and characterization methods for peptoid BCP; additional experimental details on FRET, SAXS, ANS encapsulation experiments; MALDI-TOF MS spectra, HPLC chromatograms, and analysis of synthesized peptoid BCPs; Hansen solubility parameter calculations; corrected acceptor-to-donor ratio (Icorr,DAN/INAP) plots; CMC plots; Guinier plot analysis of SAXS data; plot of micellar boundary radius (*R*_m_) for each hybrid micelle; additional ANS fluorescence emission spectra and analysis (PDF)

## #

The Molecular Foundry, Lawrence Berkeley National Laboratory, Berkeley, CA 94720, United States
