# Supplementary material for: Customizing Ionic Micelles by Dynamic Coassembly of Sequence-Defined Peptoid Block Copolymers
Source: Macromolecules. 2026 Jun 8;59(12):6739–49. doi: 10.1021/acs.macromol.5c03586 (PMC13296474; doi:10.1021/acs.macromol.5c03586)
Supplement: Supplementary file 1 [file ma5c03586_si_001.pdf]

Supporting Information

Customizing Ionic Micelles by Dynamic Co-Assembly of Sequence-Defined Peptoid Block Copolymers

Erin Tsai,[a] Meng Zhang,[a,b] Guan-Rong Huang,[c] Katelyn Hall,[a] Richard E. Gillilan,[d] Qingqiu Huang,[d] Revati Kumar,[a] and Donghui Zhang[a,*]

[a.] *Department of Chemistry and Macromolecular Studies Group, Louisiana State University, Baton Rouge, LA 70803, United States*

[b.] *Current address: The Molecular Foundry, Lawrence Berkeley National Laboratory, Berkeley, CA 94720, United States*

[c.] *Department of Engineering and System Science, National Tsing Hua University, Hsinchu 30013, Taiwan 2. Physics Division, National Center for Theoretical Sciences, Taipei 10617, Taiwan*

[d.] *MacCHESS (Macromolecular Diffraction Facility at CHESS), Cornell University, Ithaca, New York 14850, United States*

*Corresponds to dhzhang@lsu.edu

Experimental Details

Materials. All solvents were used as received unless otherwise noted. Acetic anhydride (Reagent grade), triethylamine (Reagent grade), trifluoroacetic acid (Reagent grade), tetrahydrofuran (Optima), methylene chloride (HPLC grade), *N*,*N*-dimethylformamide (ACS grade), methanol (Reagent grade), acetonitrile (Reagent grade), and *N*-methylpyrrolidone were all purchased from Fisher Scientific. Rink amide resin, bromoacetic acid (≥99%), and *N*,*N*'-diisopropylcarbodiimide (≥99%) were purchased from Chem Impex International, *n*-Decyl amine (>98%), 2-methoxyethylamine (≥98%), and 4-methylpiperidine (≥98%) were obtained from TCI. 2-(2-naphelyl)ethanamine (97%) was purchased and the dansyl chloride was purchased from Sigma Aldrich (≥99%), *β*-alanine *t*-butyl ester hydrochloride (95%) was purchased from Chem Impex International and sodium hydroxide pellets (ACS) were purchased from VWR Life Sciences.

Synthesis and Characterization of Sequence-Defined Peptoid Oligomers. Peptoid block copolymers were synthesized by a solid-phase sub-monomer method, using an adapted reported procedure (**Scheme S1**),[S1,2] on a Prelude X Peptide Synthesizer. In a general synthesis, Rink amide resin (495 μmol -NH_2, 0.67 mmol/g) was deprotected in 20% 4-methylpiperidine in *N*,*N*'-Dimethylformamide (DMF) for 12 min. The resulting amine-functionalized resin was allowed to react with bromoacetic acid in DMF (1 mL, 0.4 M) with 1 eq of *N*,*N*'- diisopropylcarbodiimide (DIC) for 20 min. Subsequently the resin was washed 5 × 20 mL with DMF. In the amine displacement step, the bromoacetylated resin was treated with the appropriate amine (*n*-decylamine, 2-methoxyethyl, or *β*-alanine *t*-butyl ester hydrochloride) in either DMF (2-methoxyethylamine and *β*-alanine *t*-butyl ester hydrochloride) or *N*-methylpyrrolidone (*n*-decylamine). A concentration of 2.67 M of amine was employed for the displacement step with *n*-decylamine and 2-methoxyethylamine and was reacted for 30 min. For the displacement step of the *β*-alanine *t*-butyl ester hydrochloride, triethylamine (6 mL, 3.6 M in DMF) was added to the reaction vessel and reacted for 90 min. Upon completion of the displacement of alanine *t*-butyl ester hydrochloride, the resin was mixed with chloroform (5 mL), followed by subsequent resin washes with DMF (10 mL), chloroform (3 × 10 mL), and DMF (3 × 20 mL). For all other monomers, the resin was only repeatedly washed with DMF (5 × 20 mL) after draining the excess amine for the displacement step. The bromoacetylation and displacement steps were repeated until the desired peptoid sequence was achieved. The peptoid-functionalized resins were resuspended in methylene chloride (DCM). The peptoids were then cleaved from the resins and deprotected using 30% trifluoroacetic acid (TFA) in DCM for 40 min. The solution was evaporated under nitrogen. Crude peptoid solids (800 mg) were then dissolved in tetrahydrofuran (THF, 12 mL) and triethylamine (TEA, 2-3 mL) and then reacted with acetic anhydride (600 mL) for 2-3 h to endcap the *N*-terminus with acetyl groups. The solution was dried by evaporation under a stream of nitrogen. The resulting peptoid solid were redissolved in deionized water and dialyzed for 2 days to remove excess TFA, TEA, and acetic anhydride. The resulting solution of peptoids was lyophilized to produce a white, fluffy powder.

The NAP-labelled peptoid block copolymers (NAP-SEQ 1, NAP-SEQ 2, and the NAP-SEQ 5) were synthesized by a similar procedure (**Scheme S2**) except that the last *N*-decyl glycine residue was replaced by a *N*-2-naphthalenylethyl glycine unit. For the bromoacetylation step, the amine-functionalized resin was treated with bromoacetic acid in DMF (1 mL, 0.4 M) with 1 eq of *N*,*N*'- diisopropylcarbodiimide (DIC) for 20 min. Subsequently, the resin was washed 5 × 20 mL with DMF. In the amine displacement step, the bromoacetylated resin was treated with 2-(2-

naphthyl)ethylamine in NMP (2.0 M) for 90 min. The resin was repeatedly washed with DMF (5 × 20 mL) and then DCM (5×10 mL). The peptoid-functionalized resins were resuspended in DCM. The peptoids were then cleaved and deprotected using 25% TFA in DCM for 40 mins. The crude peptoid solid (800 mg) were then dissolved in THF (16 mL) and TEA (2 mL) and allowed to react with acetic anhydride (600 mL) for 2-3 h to endcap the *N*-terminus with acetyl groups. The solution was dried by evaporation under a stream of nitrogen. The peptoid solids were redissolved in deionized water and dialyzed for 2 days to remove excess TFA, TEA and acetic anhydride. The resulting solution of peptoids were lyophilized for 2-3 days to produce a white, fluffy powder.

The DAN-labelled peptoids (DAN-SEQ 1, DAN-SEQ 2, and DAN-SEQ 5) were synthesized by a similar procedure (**Scheme S3**) except that the terminal *N*-decyl glycine monomer was replaced by a *N*-DAN end-capping group. In the installation of *N*-DAN end-groups, peptoid-functionalized resins (400 mg) were swelled in DCM (12 mL) and then allowed to react with TEA (280 mL, 10 eq.) and 5-(dimethylamino)naphthalene-1-sulfonyl chloride (0.4 g, 5 eq.) overnight. The resins were subsequently washed with excess THF (5 × 20 mL) and DCM (5 × 20 mL). The washed resins were resuspended in DCM. The peptoids were then cleaved and deprotected using 30% TFA in DCM for 40 mins. The solution was dried by evaporation under a stream of nitrogen. The resulting peptoid solids were redissolved in deionized water and dialyzed for 2 days to remove excess TFA. The resulting solution of peptoids was lyophilized for 2-3 days to produce a white, fluffy powder.

Matrix-Assisted Laser Desorption Ionization Time-of-Flight Mass Spectrometry (MALDI-TOF MS). MALDI-TOF MS measurements were conducted on a Bruker UltrafleXtreme tandem time-of-flight (TOF) mass spectrometer equipped with a smartbeam-II 1000 Hz laser (Bruker Daltonics, Billerica, MA). The instrument was calibrated with Peptide Calibration Standard II (Bruker Daltonics, Billerica, MA). A saturated solution of α-cyano-4-hydroxycinnamic acid (CHCA) in methanol was used as the matrix in all measurements. The polymer solution samples (1.0 mg/mL) were mixed with the saturated matrix solutions at 1:1 volume ratio mixed thoroughly. The mixtures (1 μL) were deposited onto a 384-well ground-steel sample plate and were allowed to dry in air prior to measurement using positive reflector mode. Data analysis was carried out using FlexAnalysis software and results are presented below (**Figure S1**). Experimentally determined exact molecular weights of various sequence-defined peptoid block copolymers from **Figure S1** by the MALDI-TOF MS analysis and the calculated exact molecular weights based on molecular formula were reported in **Table S1**.

High Performance Liquid Chromatography (HPLC). HPLC analysis was performed with a Waters 616 pump, Waters 2707 Autosampler, and a 996 Photodiode Assay Detector which are controlled by Waters Empower 2 Software. Separation was performed on a Waters XSelect HSS Cyano column (3.5 μm, 75 × 3 mm) by a gradient resulting from mixing eluents A (0.1% TFA in water) and B (0.1% TFA in acetonitrile). The gradient ran from 40% B to 70% B in 30 min. The flow rate was 0.4 mL/min, and the detected wavelength was 215 nm. Samples were prepared for analysis by dissolving the peptoid block copolymers (0.5 mg/mL) in a 60% water/40% acetonitrile-solvent mixture. Representative HPLC chromatograms are shown in **Figure S2**.

Förster Resonance Energy Transfer (FRET) Experiments. Stock solutions of DAN- and NAP-labeled peptoids, as well as the corresponding unlabeled peptoids, were prepared at 3.0 mg/mL by dissolving the respective polymers in preboiled ultrapure water at room temperature. The stock solutions were allowed to equilibrate overnight, and the pH was adjusted to 9.0 using 2.0

M $NaOH_{aq}$ to ensure complete ionization of the *N*-2-carboxyethyl glycine (N_{CE}) units. Subsequently, peptoid solutions containing 10.0 wt% of DAN-labeled or NAP-labeled peptoid chains (with remaining 90.0 wt% being the corresponding unlabeled peptoids) at a total peptoid concentration of 3.0 mg/mL were individually prepared from the above stock solutions. The solutions were annealed at 80 °C for 3 h, cooled to room temperature, and equilibrated overnight (>8 h). Equal volume of the above DAN- or NAP-labeled peptoid solutions was then combined and allowed to sit on the benchtop for 6 h prior to fluorescent measurement. Note that the final peptoid concentration used in FRET measurement is 3.0 mg/mL with 5.0 wt% DAN-labeled peptoid, 5.0 wt% NAP-labeled peptoid, and 90.0 wt% unlabeled peptoids. Peptoid solutions (3.0 mg/mL) containing 5.0 wt% DAN-labeled and 95.0 wt%. corresponding unlabeled peptoid (or 5.0 wt% NAP-labeled and 95.0 wt% corresponding unlabeled peptoids were prepared as controls. These controls were also annealed at 80°C for 3 h, cooled to room temperature (20°C), equilibrated overnight (>8 h) before measurement.

Stock solutions of NAP-MEO and DAN-MEO were prepared at a concentration of 3.0 mg/mL by dissolving the respective peptoid solids in preboiled ultrapure at room temperature. The stock solutions were allowed to stand at room temperature overnight (>8 h). The solution pH was then adjusted to 9.0 using a 2.0 M $NaOH_{aq}$ solution. The stock solutions of NAP-MEO (3.0 mg/mL) and DAN-MEO (3.0 mg/mL) was individually diluted and then mixed at 1:1 molar ratio of NAP-MEO: DAN-MEO to arrive at a series of solutions (3 mL) with a total peptoid concentrations of 0.75, 0.50, 0.375, 0.25, 0.125, and 0.0625 mg/mL, respectively. Samples were then heated at 80°C for 3 h. The samples were allowed to stand at room temperature (20°C) overnight before fluorescence emissions measurement.

Fluorescence emission spectra of all solution samples were collected between 300 and 800 nm at 20°C using an excitation wavelength of 289 nm, 2.0 nm excitation and emission slit widths, a step size of 1.0 nm, and an integration time of 0.1 s per point. Fluorescence spectra were acquired from a single sample across three repeated measurements, with duplicate scans collected for each measurement to assess reproducibility. For data analysis, the mean fluorescence intensity and associated standard deviation were calculated at each wavelength from six independent measurements. The FRET efficiency (E) calculation was performed with the donor fluorescence intensities obtained from the emission spectra at the donor emission maximum (λ=336 nm) before and after mixing. The calculation for the corrected acceptor-to-donor ratio was performed with the donor emissions intensity obtained from the maximum (λ=336 nm) of the donor emissions signal after mixing and the acceptor emissions (λ=518 nm) before and after mixing.

Critical Micelle Concentration (CMC) Determination. Stock solutions of SEQ 1, SEQ 2, and SEQ 5 were prepared at a concentration of 3.0 mg/mL by dissolving the respective peptoid block copolymers at room temperature in 60 mM NaCl solution made from preboiled ultrapure water. Stock solutions were allowed to stir overnight on the benchtop (> 8 h). Subsequently the binary mixtures of SEQ 1/SEQ 5, SEQ 2/SEQ 5, and SEQ 2/SEQ 1 at the 1:0, 2:8, 4:6, 6:4, 8:2, and 0:1 molar ratio (three separate replicates) were prepared from the stock solutions of SEQ 1, SEQ 2, and SEQ 5. The solution pH was adjusted to 9.0 using an aqueous NaOH solution (1.0 M) in 60 mM NaCl. The solution samples were then heated at 80°C for 3 h, cooled to room temperature, and equilibrated overnight (>8 h). Peptoid solutions with 1.5, 0.5, 0.25, 0.10, 0.05, 0.01, 0.005, and 0.001 mg/mL concentrations were prepared by serial dilution. Using the static light-scattering method,[S3-S5] the absolute count rate was measured for each solution for

approximately 3 min with an equilibration time of 2.0 min between each measurement (three replicates) at a constant back scattering angle of 175°.

Small-Angle X-Ray Scattering (SAXS) Experiments. Stock solutions of SEQ 1, SEQ 2, and SEQ 5 were individually prepared at 3.0 mg/mL concentration by dissolving the respective peptoid block copolymers in 60 mM NaCl solution (made from preboiled ultrapure water) at room temperature. The stock solutions were stirred at room temperature overnight (≥ 8 h). Solutions containing the binary mixtures of SEQ 1/SEQ 5, SEQ 2/SEQ 5, and SEQ 2/SEQ 1 at 1:0, 2:8, 4:6, 6:4, 8:2, and 0:1 molar ratio were prepared using the stock solutions of SEQ 1, SEQ 2, and SEQ 5. The solution pH was adjusted to 9.0 using an aqueous NaOH solution (1.0 M) in a 60 mM NaCl. The solution samples were then annealed at 80°C for 3 h, cooled to room temperature, and filtered through 0.45 mm PES springe filters. SAXS measurements of the sequence-defined peptoid micellar solutions were conducted at the Cornell High Energy Synchrotron Source (CHESS, Ithaca, NY) on the ID7A1 Bio-SAXS beamline SAXS instrument equipped with an EIGER 4M detector using an X-ray wavelength of λ = 1.25 Å and covering q-range between 0.008 $Å^{-1}$ and 0.5 $Å^{-1}$. Sample solutions were loaded into a quartz capillary (diameter = 1.5 mm, wall thickness = 0.01 mm) flow-cell kept at 20 ± 0.1 °C for SAXS measurement. Fifty images for each peptoid micellar solution samples and background solutions (*i.e.*, 60 mM NaCl in H_2O at pH = 9.0) were measured. The solvent and coherent background (60 mM NaCl aqueous solutions at pH=9.0) were subtracted via RAW software,[S6] and SasView software[S7] (http://www.sasview.org/) was used for further data analysis.

ANS Experiments. Stock solutions of SEQ 1- SEQ 5 were prepared at a concentration of 3.0 mg/mL by dissolving the respective peptoid block copolymers in preboiled ultrapure water at room temperature. The prepared stock solutions were allowed to stand at room temperature overnight. A stock solution of ANS (200 mM) was prepared and adjusted to pH=9.0 using an aqueous NaOH solution (2.0 M). Subsequently the binary mixtures of SEQ 1: SEQ 5, SEQ 2: SEQ 5, SEQ 3: SEQ 5, SEQ 4: SEQ 5, SEQ 1: SEQ 2, and SEQ 3:SEQ 4 at molar ratios of 1:0, 2.5:7.5, 5.0:5.0, 7.5:2.5, and 0:1 (three replicates) were prepared. A measured volume of the ANS stock solution (200 mM) was added so that the final ANS concentration was 20.0 mM and the final peptoid concentration was 2.8 mg/mL. Each stock solution was annealed at 80°C for 3 h and cooled to room temperature overnight before fluorescence measurement. Fluorescence emission of solutions containing peptoids and ANS was measured between 360 nm and 800 nm at 20°C using an excitation wavelength of 350 nm, 2.0 nm excitation and emission slit widths, a step size of 1.0 nm, and an integration time of 0.1 s per point. Three replicates of each solution sample were measured two times (6 measurements total). For data analysis, the mean fluorescence intensity and associated standard deviation were calculated at each wavelength from six independent measurements. All average spectra were normalized to the maximum fluorescence intensity of SEQ 5, which exhibited the highest emission, to obtain relative fluorescence intensities.

Scheme S1. Representative synthesis of SEQ 1 by the sub-monomer method and subsequent off-resin end-capping the *N*-terminus with acetyl groups.

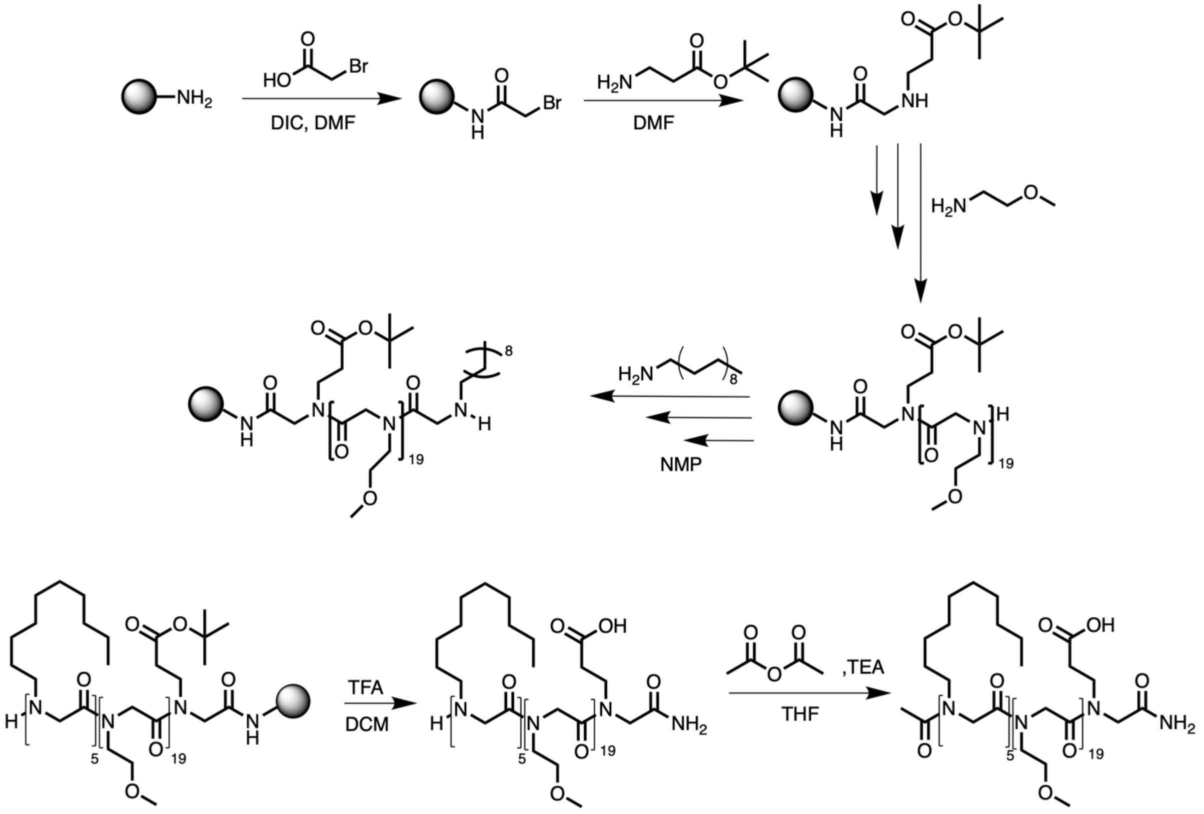

Scheme S2. Representative synthesis of NAP-SEQ 1 by the sub-monomer method and subsequent off-resin end-capping the *N*-terminus with acetyl groups.

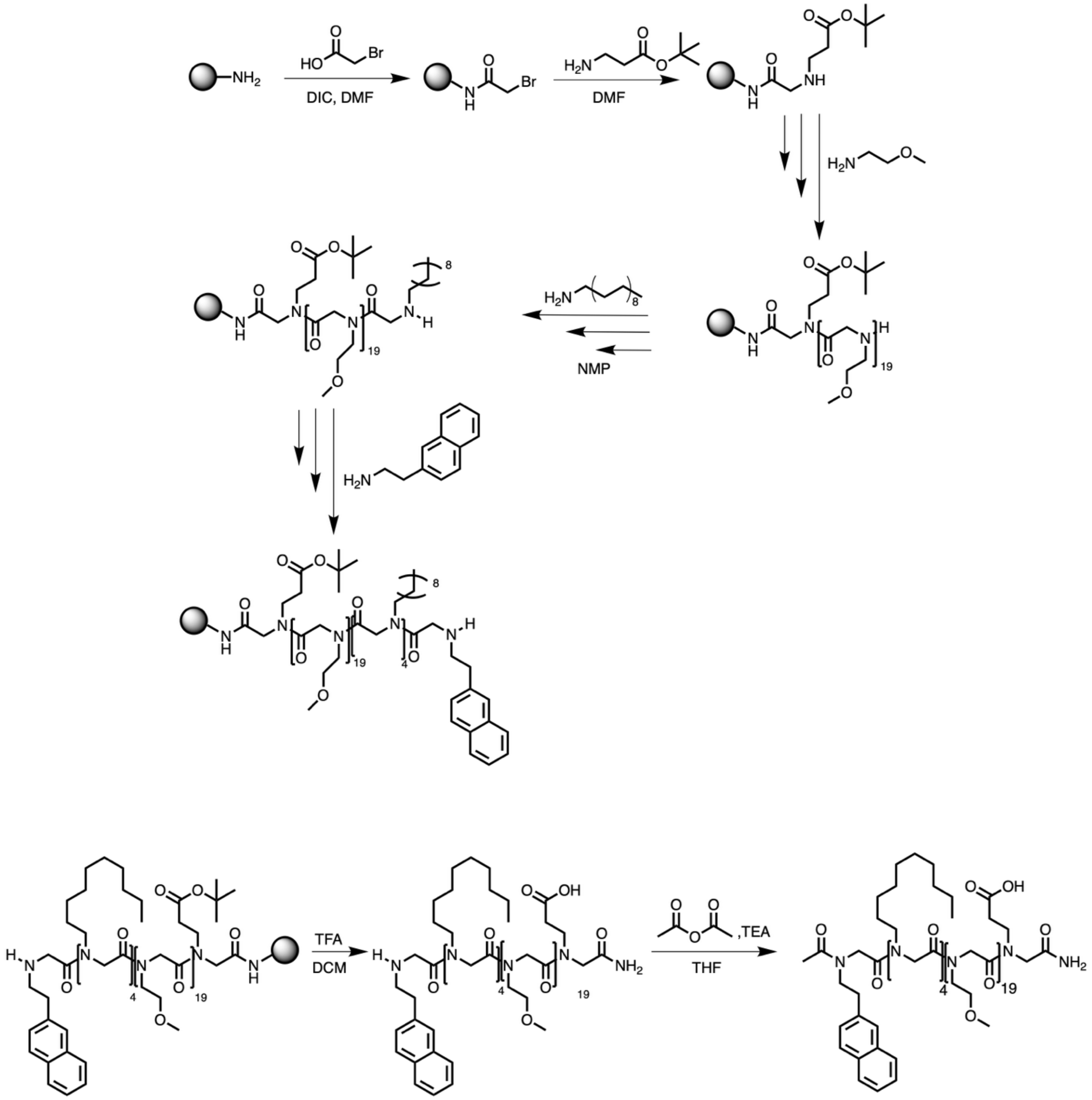

Scheme S3. Representative synthesis of DAN-SEQ 1 by the sub-monomer method and subsequent off resin end-capping of the *N*-terminus with a DAN group.

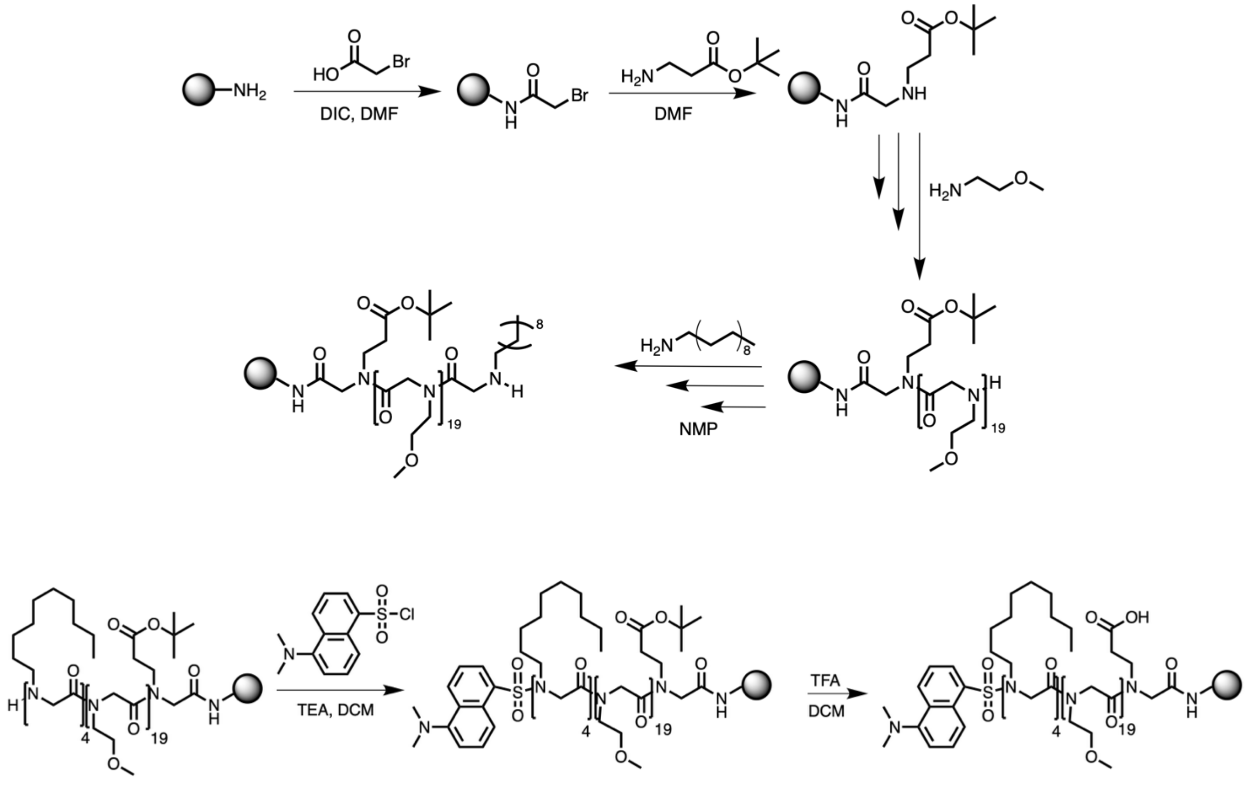

MALDI-TOS MS Spectra.

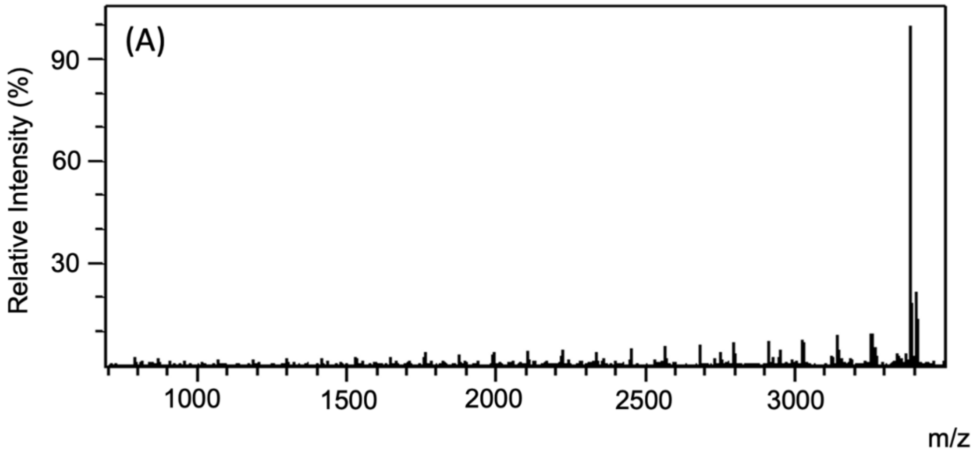

Figure S1.A. Representative MALDI-TOF MS spectrum for SEQ 1.

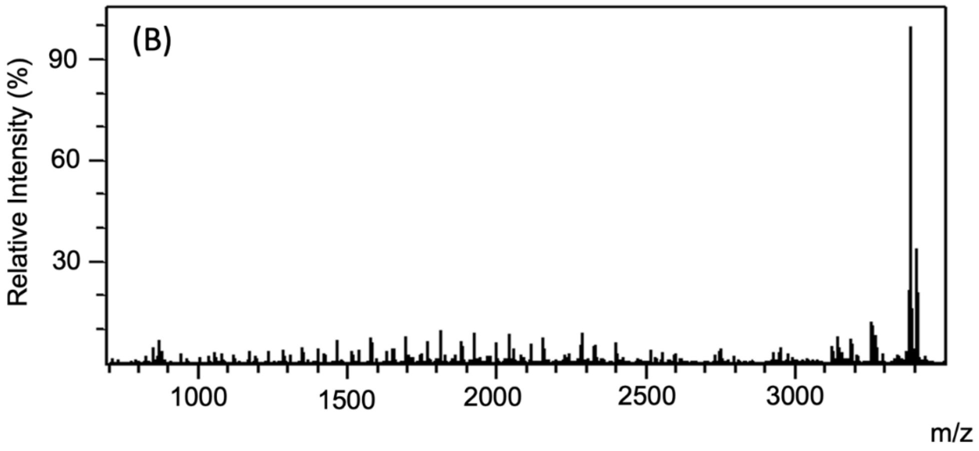

Figure S1.B. Representative MALDI-TOF MS spectrum for SEQ 2.

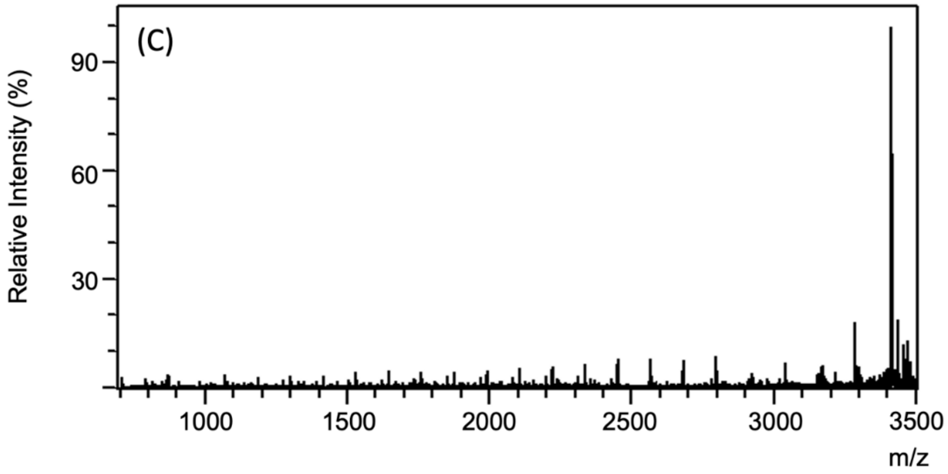

Figure S1.C. Representative MALDI-TOF MS spectrum for SEQ 3.

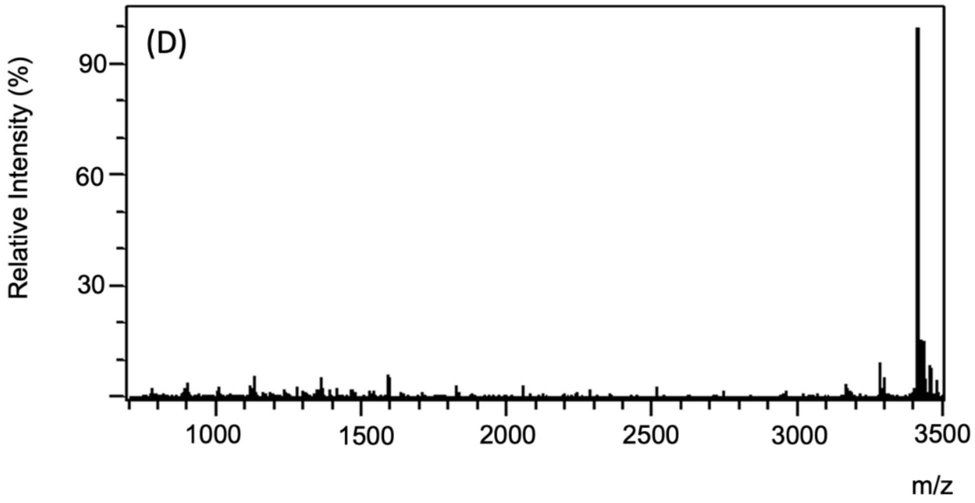

Figure S1.D. Representative MALDI-TOF MS spectrum for SEQ 4.

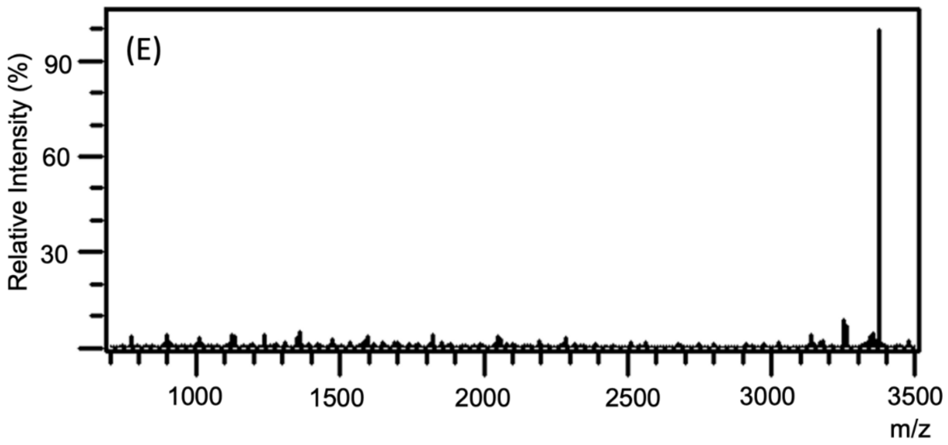

Figure S1.E. Representative MALDI-TOF MS spectrum for SEQ 5.

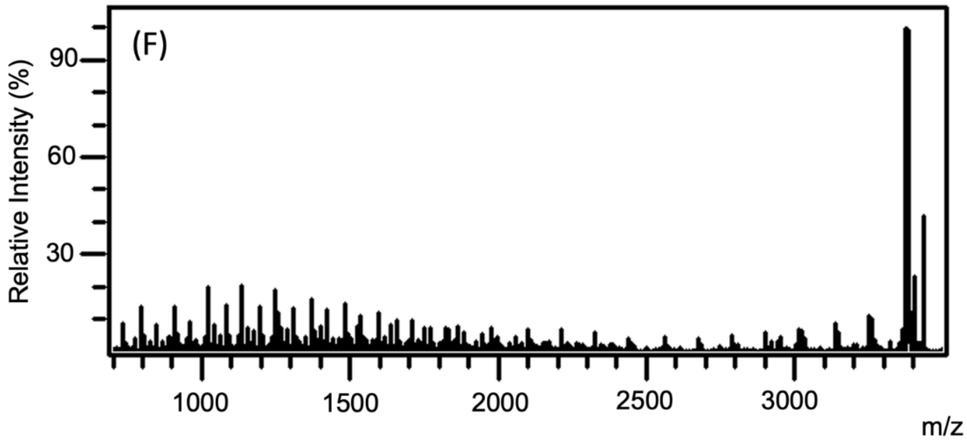

Figure S1.F. Representative MALDI-TOF MS spectrum for DAN-SEQ 1.

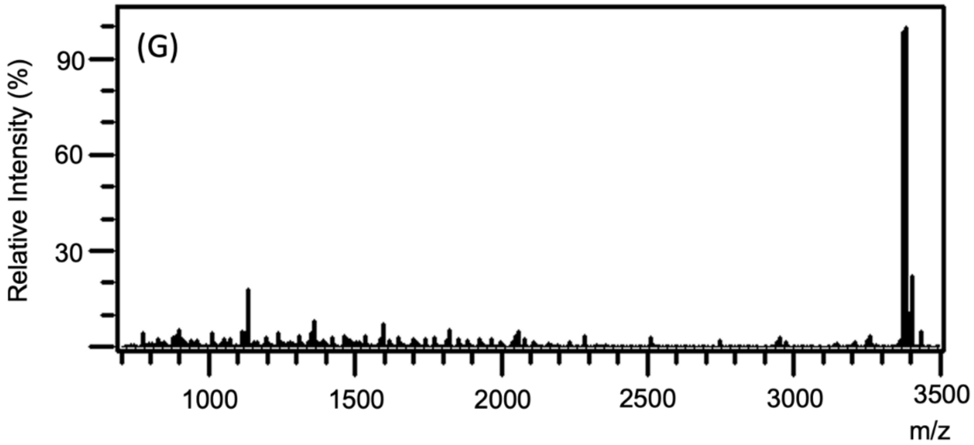

Figure S1.G. Representative MALDI-TOF MS spectrum for DAN-SEQ 2

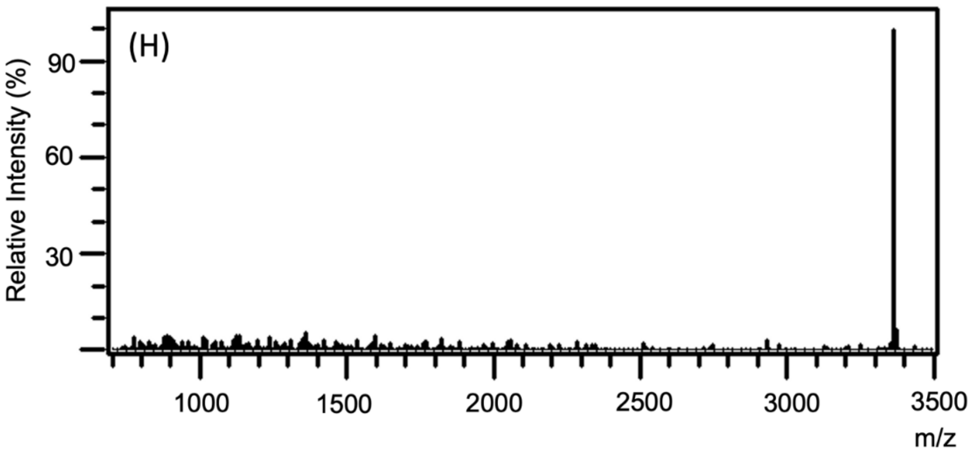

Figure S1.H. Representative MALDI-TOF MS spectrum for DAN-SEQ 5.

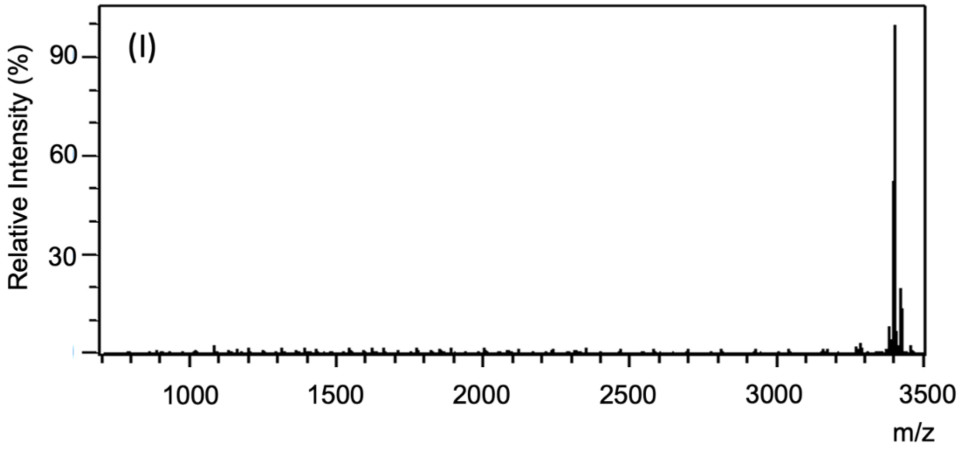

Figure S1.I. Representative MALDI-TOF MS spectrum for NAP-SEQ 1.

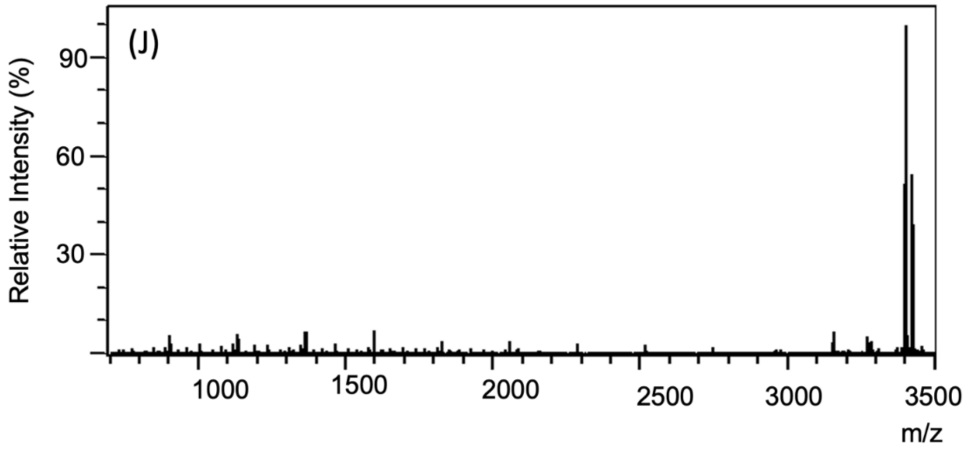

Figure S1.J. Representative MALDI-TOF MS spectrum for NAP-SEQ 2.

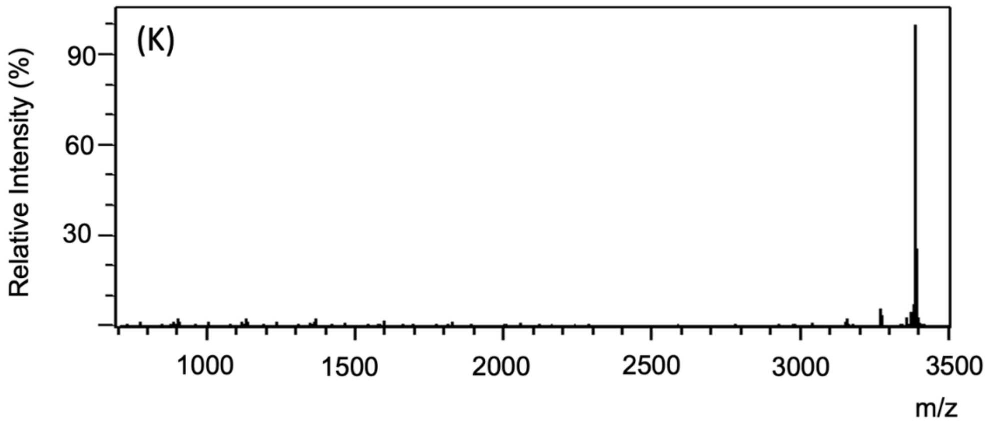

Figure S1.K. Representative MALDI-TOF MS spectrum for NAP-SEQ 5.

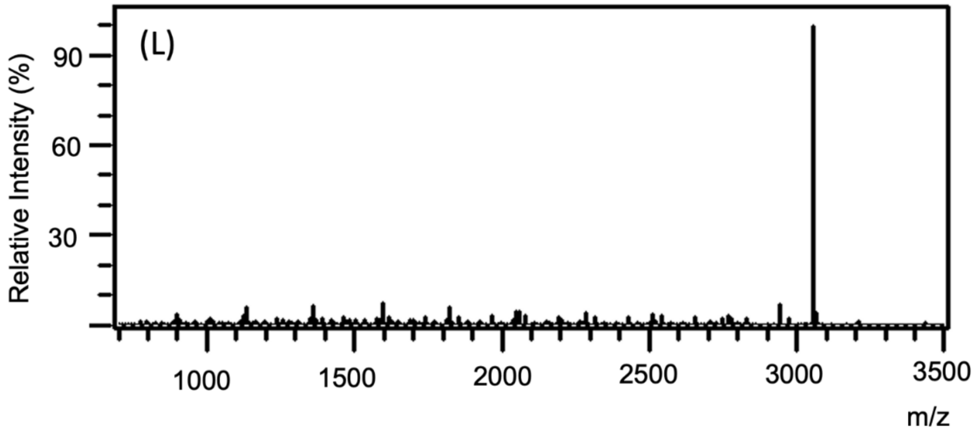

Figure S1.L. Representative MALDI-TOF MS spectrum for NAP-MEO.

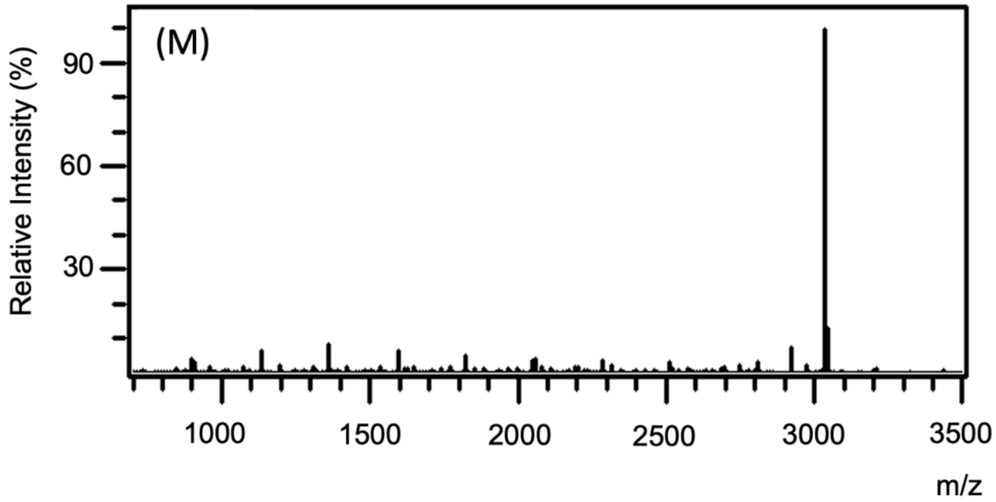

Figure S1.M. Representative MALDI-TOF MS spectrum for DAN-MEO.

HPLC Chromatograms

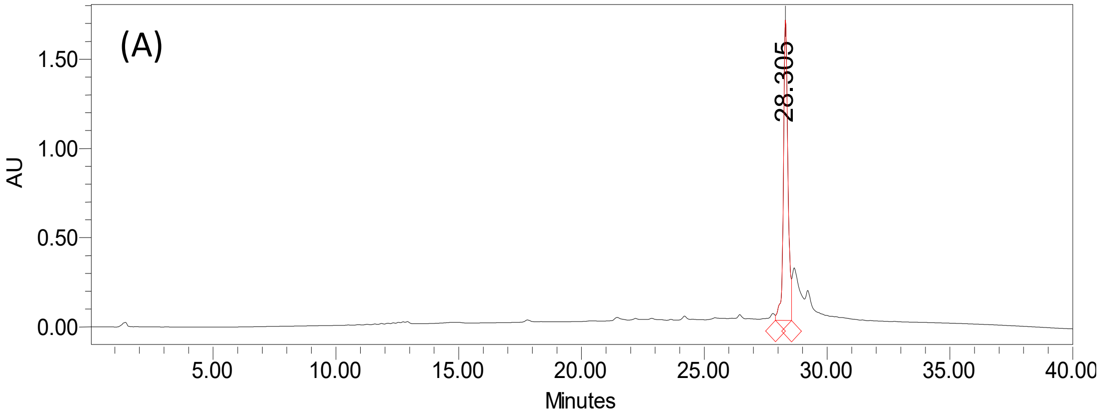

Figure S2.A. Representative HPLC chromatogram for SEQ 1.

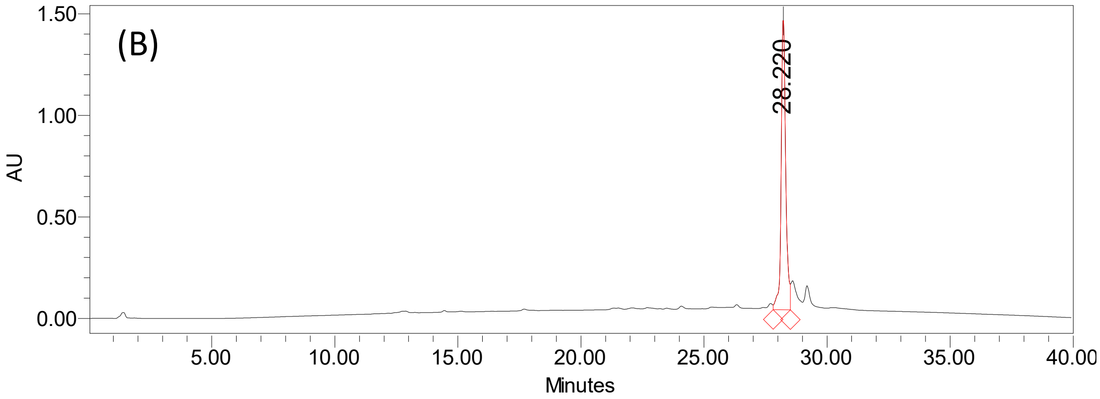

Figure S2.B. Representative HPLC chromatogram for SEQ 2.

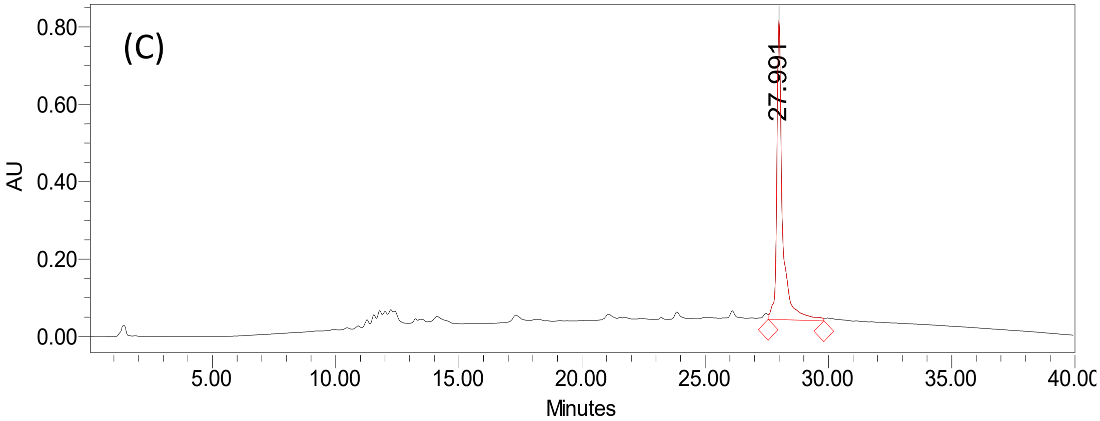

Figure S2.C. Representative HPLC chromatogram for SEQ 3.

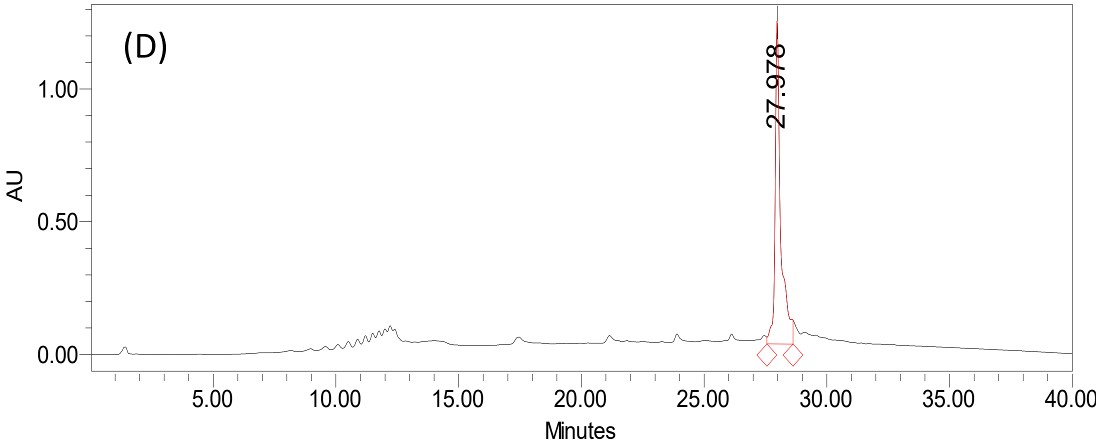

Figure S2.D. Representative HPLC chromatogram for SEQ 4.

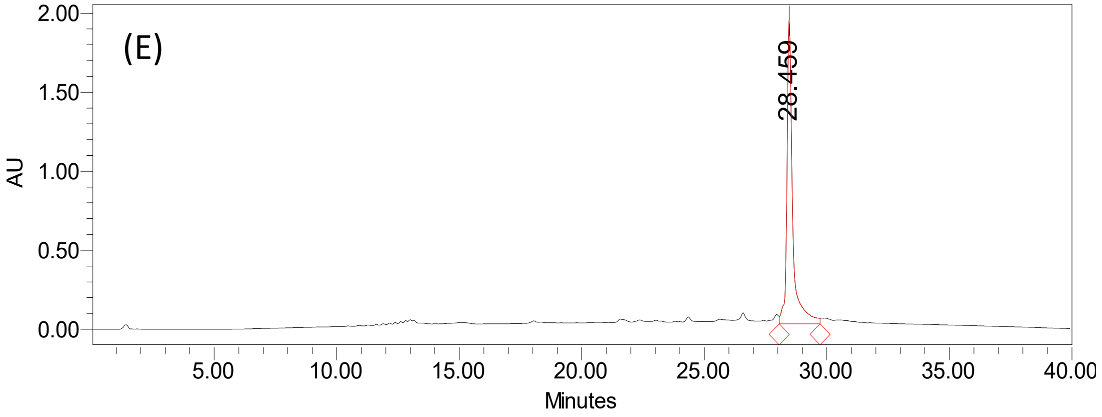

Figure S2.E. Representative HPLC chromatogram for SEQ 5.

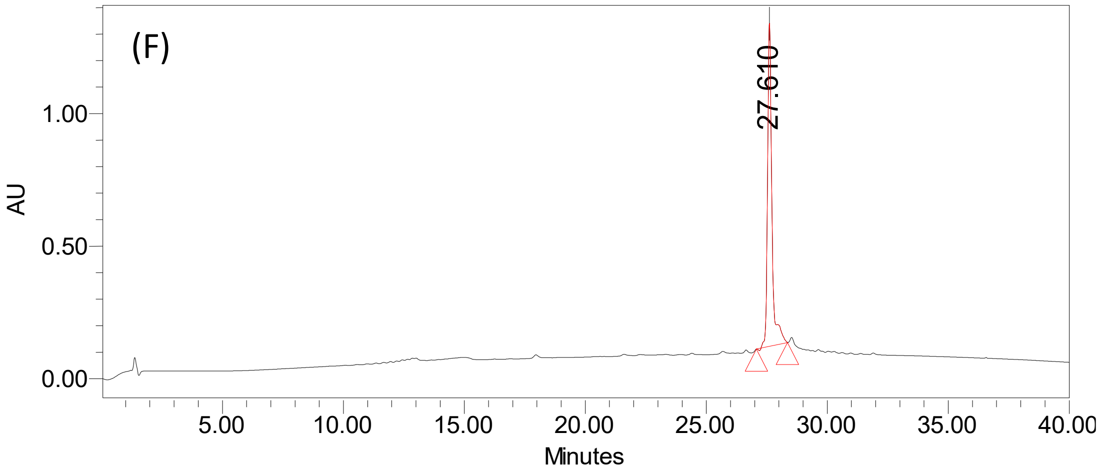

Figure S2.F. Representative HPLC chromatogram for NAP-SEQ 1.

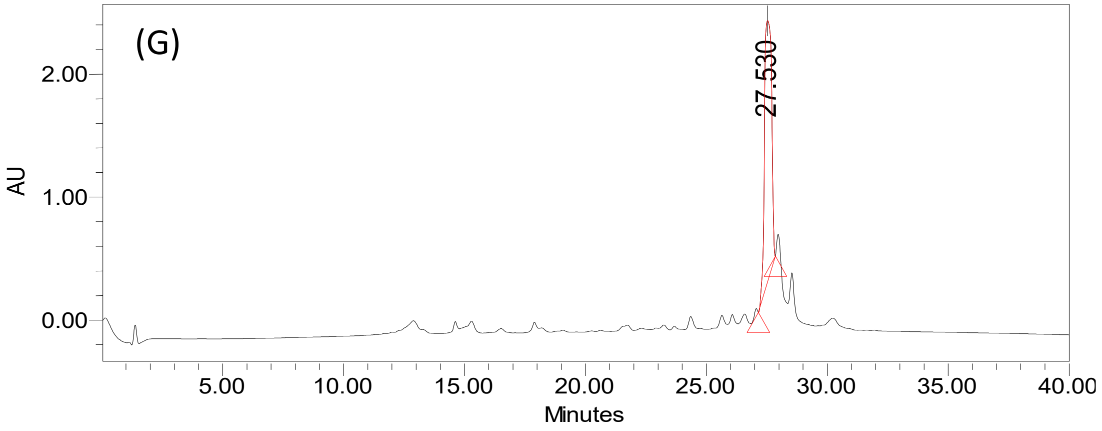

Figure S2.G. Representative HPLC chromatogram for NAP-SEQ 2.

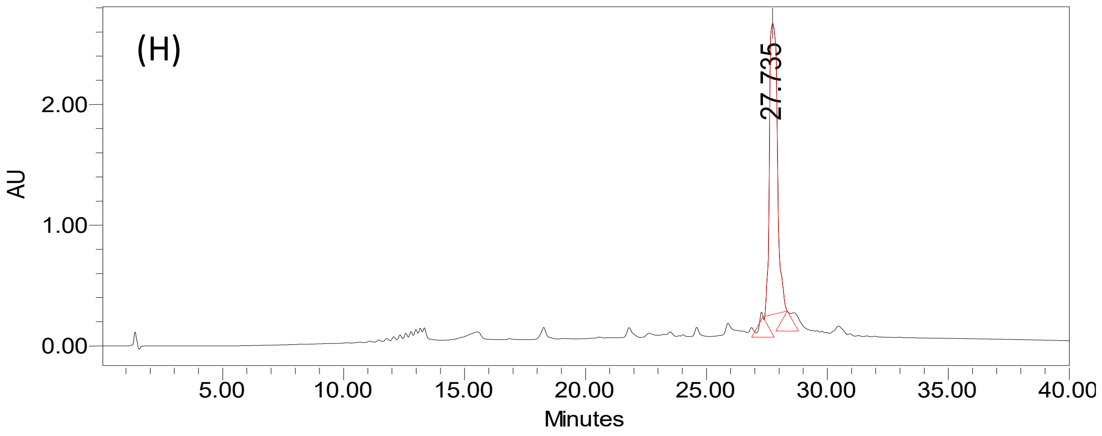

Figure S2.H. Representative HPLC chromatogram for NAP-SEQ 5.

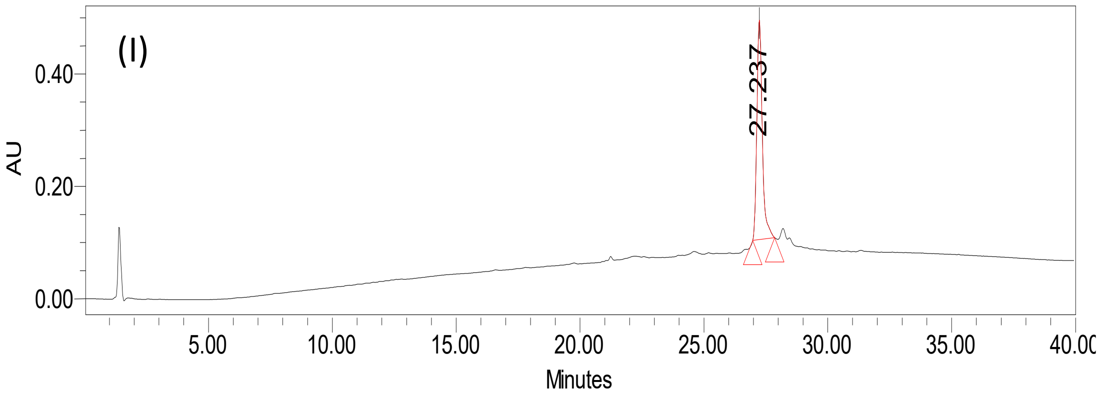

Figure S2.I. Representative HPLC chromatogram for DAN-SEQ 1.

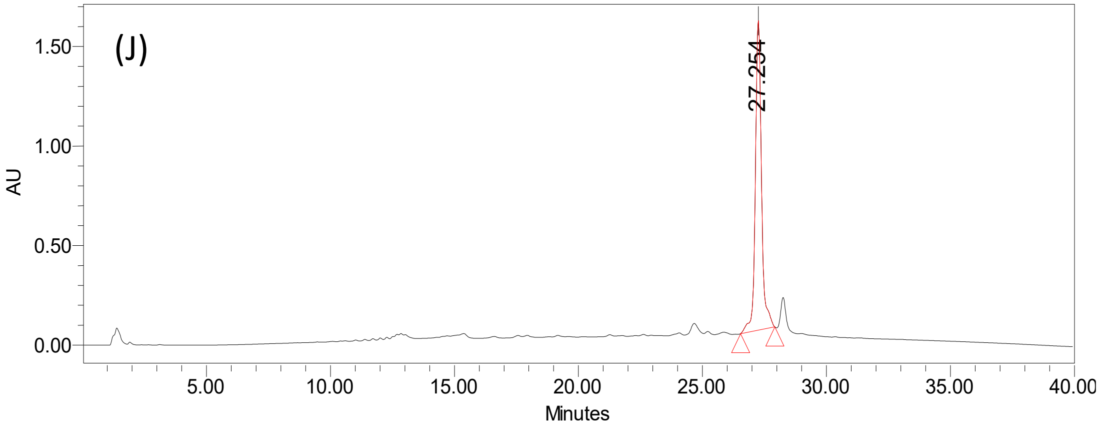

Figure S2.J. Representative HPLC chromatogram for DAN-SEQ 2.

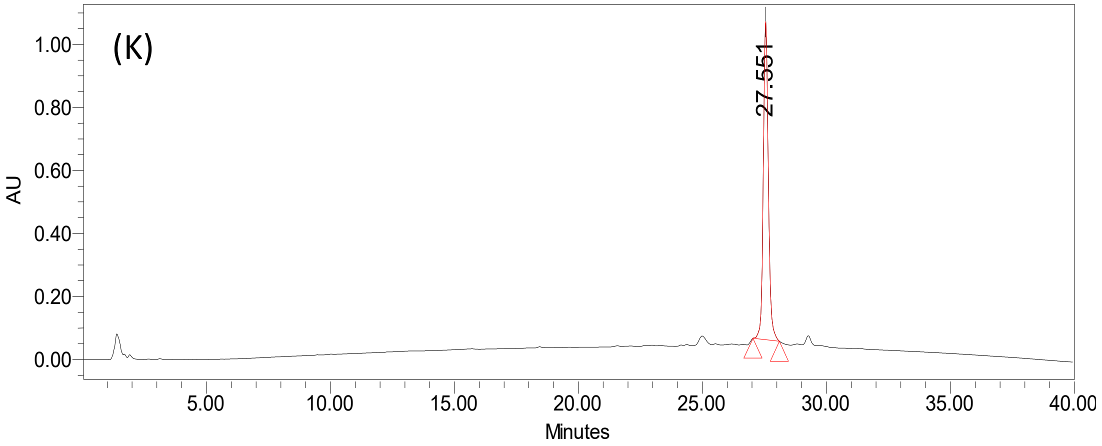

Figure S2.K. Representative HPLC chromatogram for DAN-SEQ 5.

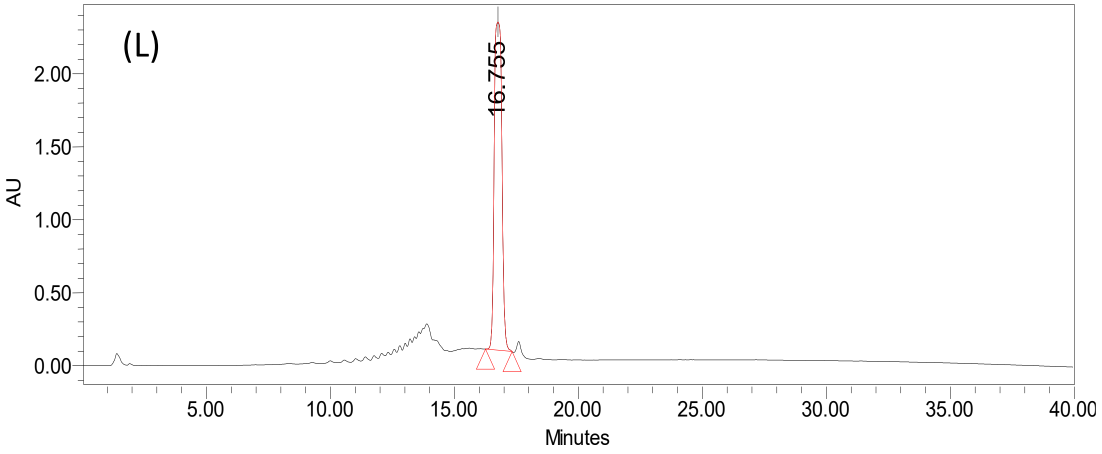

Figure S2.L. Representative HPLC chromatogram for NAP-MEO.

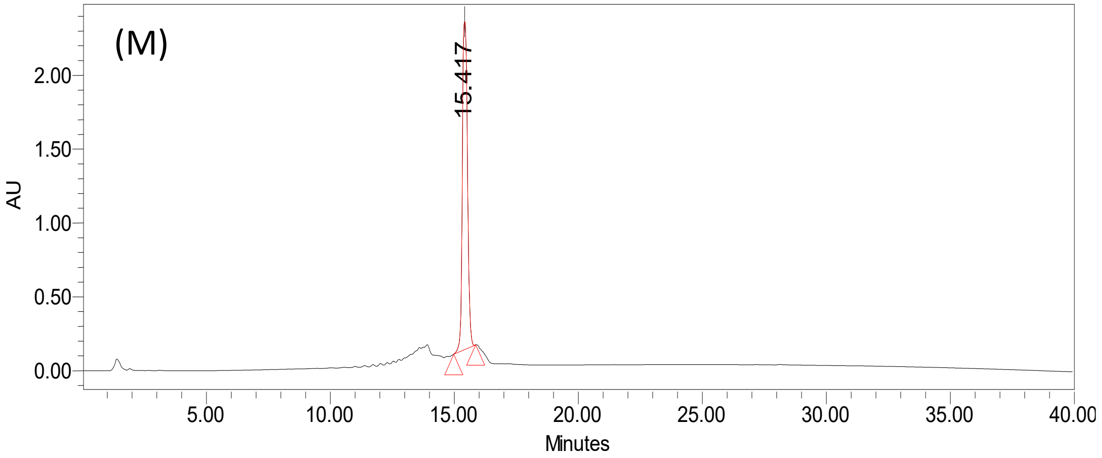

Figure S2.M. Representative HPLC chromatogram for DAN-MEO.

Table S1. Experimentally determined exact molecular weights of various sequence-defined peptoid BCP, **Figure S1**) by the MALDI-TOF MS analysis, the calculated exact molecular weights based on molecular formula and the sample purity level based on HPLC analysis.

Sequence	Molecular Formula		Calc. (m/z)	Found (m/z)	Purity (%)
SEQ 1	$C_{162}H_{298}N_{26}O_{47}Na^{+}$<br>$C_{162}H_{297}N_{26}O_{47}Na^{+}$	[M+Na] $^{+}$<br>[M+2Na-H]$^{+}$	3383.16<br>3404.15	3383.05<br>3405.09	96
SEQ 2	$C_{162}H_{298}N_{26}O_{47}Na^{+}$<br>$C_{162}H_{298}N_{26}O_{47}K^{+}$<br>$C_{162}H_{297}N_{26}O_{47}Na_{2}^{+}$	[M+Na]$^{+}$<br>[M+K]$^{+}$<br>[M+2Na-H]$^{+}$	3383.16<br>3399.14<br>3404.15	3381.15<br>3398.13<br>3404.16	95
SEQ 3	$C_{162}H_{294}N_{26}O_{49}Na^{+}$<br>$C_{162}H_{293}N_{26}O_{49}Na_{2}^{+}$<br>$C_{162}H_{292}N_{26}O_{49}Na_{3}^{+}$	[M+Na]$^{+}$<br>[M+2Na-H]$^{+}$<br>[M+3Na-2H]$^{+}$	3411.12<br>3433.11<br>3455.10	3410.37<br>3432.35<br>3454.32	86
SEQ 4	$C_{162}H_{294}N_{26}O_{49}Na^{+}$<br>$C_{162}H_{294}N_{26}O_{49}K^{+}$<br>$C_{162}H_{293}N_{26}O_{49}Na_{2}^{+}$<br>$C_{162}H_{292}N_{26}O_{49}Na_{3}^{+}$<br>$C_{162}H_{293}N_{26}O_{49}K_{2}^{+}$	[M+Na]$^{+}$<br>[M+K]$^{+}$<br>[M+2Na-H]$^{+}$<br>[M+3Na-2H]$^{+}$<br>M+2K-H]$^{+}$	3411.12<br>3427.09<br>3433.11<br>3455.10<br>3665.05	3411.26<br>3425.27<br>3433.25<br>3455.27<br>3465.49	87
SEQ 5	$C_{162}H_{300}N_{26}O_{46}$	[M+Na]$^{+}$	3369.18	3369.78	96
DAN-SEQ 1	$C_{160}H_{284}N_{26}O_{47}Na^{+}$	[M+Na]$^{+}$	3377.02	3376.83	97
DAN-SEQ 2	$C_{160}H_{284}N_{26}O_{47}Na^{+}$	[M+Na]$^{+}$	3377.02	3377.28	89
DAN-SEQ 5	$C_{160}H_{286}N_{26}O_{46}SNa^{+}$	[M+Na]$^{+}$	3363.05	3363.16	87
NAP-SEQ 1	$C_{164}H_{288}N_{26}O_{47}Na^{+}$<br>$C_{164}H_{287}N_{26}O_{47}Na_{2}^{+}$	[M+Na]$^{+}$<br>[M+2Na−H]$^{+}$	3397.08;<br>3419.07	3397.70;<br>3420.67	91
NAP-SEQ 2	$C_{164}H_{288}N_{26}O_{47}Na^{+}$<br>$C_{164}H_{287}N_{26}O_{47}Na_{2}^{+}$	[M+Na]$^{+}$<br>[M+2Na−H]$^{+}$	3397.08;<br>3419.07	3397.57<br>3419.57	86
NAP-SEQ 5	$C_{164}H_{290}$N26O46Na^{+}	[M+Na]$^{+}$	3360.12	3383.75	98
NAP-MEO	$C_{136}H_{234}N_{26}O_{50}Na^{+}$	[M+Na]$^{+}$	3054.11	3054.99	88
DAN-MEO	$C_{132}H_{230}N_{26}O_{50}SNa^{+}$	[M+Na]$^{+}$	3034.59	3035.14	91

Calculation of Hanson Solubility Parameters (HSPs).

The Hansen solubility parameters (HSPs) were calculated for the single *N*-2-naphthalenylethyl glycine (NAP), 5-(*N*,*N*-dimethylamino)naphthalene-1-sulfonyl (DAN), and *N*-decyl glycine residue, respectively. The HSPs contain three distinct components: the dispersion parameter (δ_d), the polarity parameter (δ_p), and another parameter to account for hydrogen bonding (δ_h). These parameters can be estimated using group contribution parameters that account for the respective molecular forces and molar volume as follows:[S8, S9]

$$\delta_d = \frac{\sum F_{di}}{V_m} \qquad \text{Eq. S1}$$

$$\delta_p = \frac{\sqrt{\sum F_{pi}^2}}{V_m} \qquad \text{Eq. S2}$$

$$\delta_h = \sqrt{\frac{\sum E_{hi}}{V_m}} \qquad \text{Eq. S3}$$

$$V_m = \sum_i n_i V_i \qquad \text{Eq. S4}$$

Where V_m is the total molecular volume contribution of the molar group i to the molar volume in the liquid state and n_i is its occurrence within the molecule, (Eq. S4).[S8, S9] Properties of polymers: their correlation with chemical structure; their numerical estimation and prediction from additive group contributions F_{di}, F_{pi}, and E_{hi} are the contributions to cohesive energy from dispersion forces, polarity, and hydrogen bonds of a structural group i. The total Hansen solubility parameter δ_T is then given by the sum of the partial parameters (Eq.S5):[S9]

$$\delta_T = \sqrt{\delta_d^2 + \delta_p^2 + \delta_h^2} \qquad \text{Eq. S5}$$

The molecular structure of the N_{NAP} peptoid monomer was dissected into five different groups: -CH_3, -CH_2-, -N<, -CO-, and -C_6H_5 (**Table S2A**). The molecular structure of the DAN N-terminal cap was dissected into five different groups: -CH_3, -N<, -SO_2-, and -C_6H_5 (**Table S2B**) On the other hand, the molecular structure of N_{DE} was broken apart into five different groups: -CH_3, -CH_2-, -CO-, and -N< (**Table S2C**). The values of F_d, F_p, and E_h for each group were obtained from the references.[S9, S10] The solubility parameters δ_d, δ_p, δ_h, and δ_T of the N_{NAP} peptoid monomer, the DAN N-terminal cap, and N_{DE} were then calculated using Eqs. S1-S5.

Table S2A. Calculation of HSPs and molar volume for NAP residue according to the Hoftyzer-Van Krevelen method.[S9]

Group	Frequency	F_{di} ($J^{1/2}$ $cm^{3/2}$ mol^{-1})	F_{pi} ($J^{1/2}$ $cm^{3/2}$ mol^{-1})	E_{hi} (J/mol)	V_m (cm^3/mol)
-CH_3	1	420	0	0	33.5
-CH_2-	3	810	0	0	48.3
-CO-	2	580	1540	4000	21.6
-C_6H_5	2	2540	220	0	104.8
-N<	1	20	800	5000	-9.0
δ_d	21.9 $MPa^{1/2}$				

δ_p	6.8 MPa$^{1/2}$				
δ_h	6.7 MPa$^{1/2}$				
δ_T	23.9 MPa$^{1/2}$				
V_m	199.2 cm^3/mol				

Table S2B. Calculation of HSPs and molar volume for the DAN group according to the Hoftyzer-Van Krevelen method.[S9]

Group	Frequency	F_{di} (J$^{1/2}$ cm$^{3/2}$ mol^{-1})	F_{pi} (J$^{1/2}$ cm$^{3/2}$ mol^{-1})	E_{hi} (J/mol)	V_m (cm^3/mol)
$-CH_3$	2	840	0	0	67.0
$-SO_2-$	1	1129	1358	11670	51.0
$-C_6H_5$	2	2540	220	0	104.8
-N<	1	20	800	5000	-9.0
δ_d	21.2 MPa$^{1/2}$				
δ_p	7.4 MPa$^{1/2}$				
δ_h	8.8 MPa$^{1/2}$				
δ_T	24.1 MPa$^{1/2}$				
V_m	213.8 cm^3/mol				

Table S2C. Calculation of HSPs and molar volume for the N_{DE} residue according to the Hoftyzer-Van Krevelen method.[S9]

Group	Frequency	F_{di} (J$^{1/2}$ cm$^{3/2}$ mol^{-1})	F_{pi} (J$^{1/2}$ cm$^{3/2}$ mol^{-1})	E_{hi} (J/mol)	V_m (cm^3/mol)
$-CH_3$	2	840	0	0	67.0
$-CH_2-$	10	2700	0	0	161.0
-CO-	2	580	1540	4000	21.6
-N<	1	20	800	5000	-9.0
δ_d	17.2 MPa$^{1/2}$				
δ_p	5.6 MPa$^{1/2}$				
δ_h	6.1 MPa$^{1/2}$				
δ_T	19.1 MPa$^{1/2}$				
V_m	240.6 cm^3/mol				

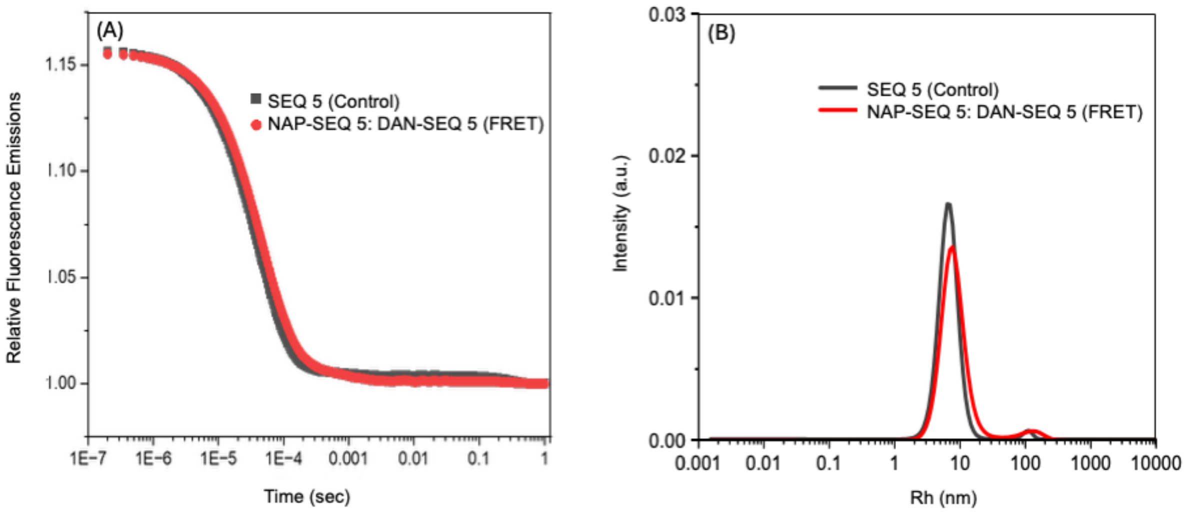

Figure S3. Dynamic light scattering (DLS) characterization of selected sequence-defined peptoid micelle and their counterparts consisting of a small fraction of fluorophore-labelled sequences. (A) DLS autocorrelation functions of SEQ 5 micelles and (B) the corresponding intensity-weighted size distribution obtained by fitting the DLS data with maximum entropy method (MEM). The SEQ 5 micelles (black line) exhibited similar size distribution as the counterparts consisting of 5 wt.% DAN-SEQ 5, 5 wt.% NAP-SEQ 5, and 90 wt.% unlabeled SEQ 5 (red), indicating that incorporation of fluorophore-labeled sequences in low content does not significantly alter the micellar structure.

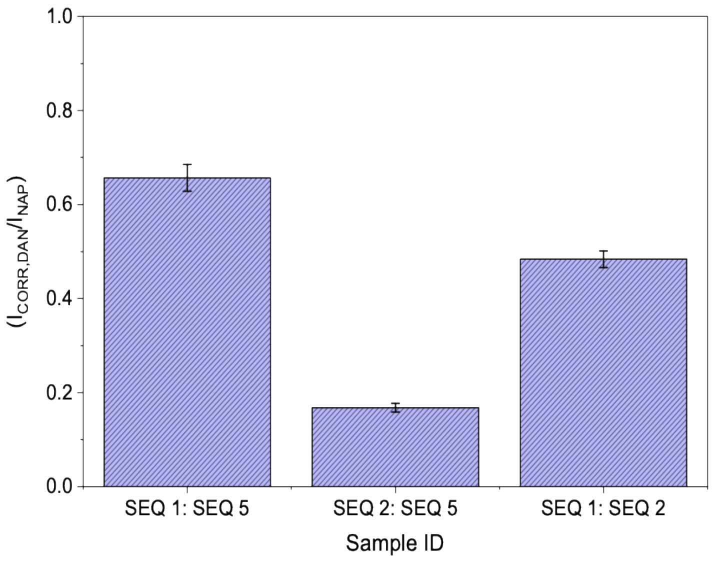

igure S4. The corrected acceptor-to-donor ratio ($I_{corr,DAN}/I_{NAP}$) defined by Eq. S6 from the fluorescence emission spectra of hybrid micellar solutions consisting of two different sequence-defined peptoid BCP (SEQ 1: SEQ 5, SEQ 2: SEQ 5, SEQ 1: SEQ 5, SEQ 2: SEQ 5, SEQ 1: SEQ 2) in 1:1 molar ratio (pH = 9.0, 20 °C, λ_{ex}= 289 nm).

Note: The corrected acceptor-to-donor ratio ($I_{corr,\ DAN}/I_{NAP}$), is defined by Eq. S6 as follows:[S11]

$$I_{\mathrm{corr,DAN}}/I_{\mathrm{NAP}} = [(I_{\mathrm{DAN}} - I_{\mathrm{DAN,0}})]/I_{\mathrm{NAP}} \quad \text{Eq. S6}$$

where I_{DAN} is the emissions intensity of the acceptor signal after mixing, I_{NAP} is the emissions intensity of the donor signal after mixing, and $I_{\mathrm{DAN,0}}$ is the emissions intensity of the acceptor only signal before mixing. Due to minimal spectral overlap between donor (NAP) and acceptor (DAN) emission, donor bleed-through into the acceptor channel was negligible. FRET was therefore quantified using a ratiometric sensitized-emission approach, where the acceptor emission under donor excitation was background-corrected using acceptor-only controls and normalized to donor emission. This ratio reflects the degree of acceptor emission enhancement relative to donor signal. It differs from the FRET efficiency parameter (E) define by Eq. 1 which only captures the donor intensity change upon mixing. Thus, corrected acceptor-to-donor ratio provides additional insight into the extent of energy transfer between donor/acceptor-tagged peptoid chains. While corrected acceptor-to-donor ratio is not a true FRET efficiency (E, Eq. 1), it serves as a semi-quantitative measure of the donor-acceptor FRET efficiency and an indicator of molecular proximity of the donor/acceptor-labelled peptoid BCP chains, assuming instrumental internal consistency and the photophysical properties of the employed donor and acceptor fluorophores remaining unchanged.

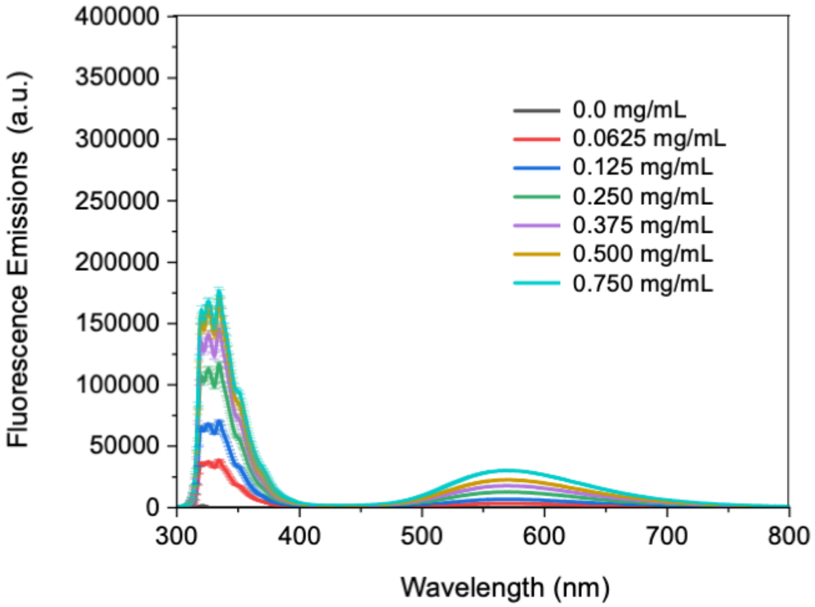

Figure S5. Fluorescence emission spectra of aqueous solutions containing NAP-MEO and DAN-MEO at 1:1 molar ratio and different total peptoid concentrations (0.0625 – 0.75 mg/mL) (pH = 9.0, 20 °C, λ_{ex}= 289 nm). Error bars represent standard deviation from six measurements.

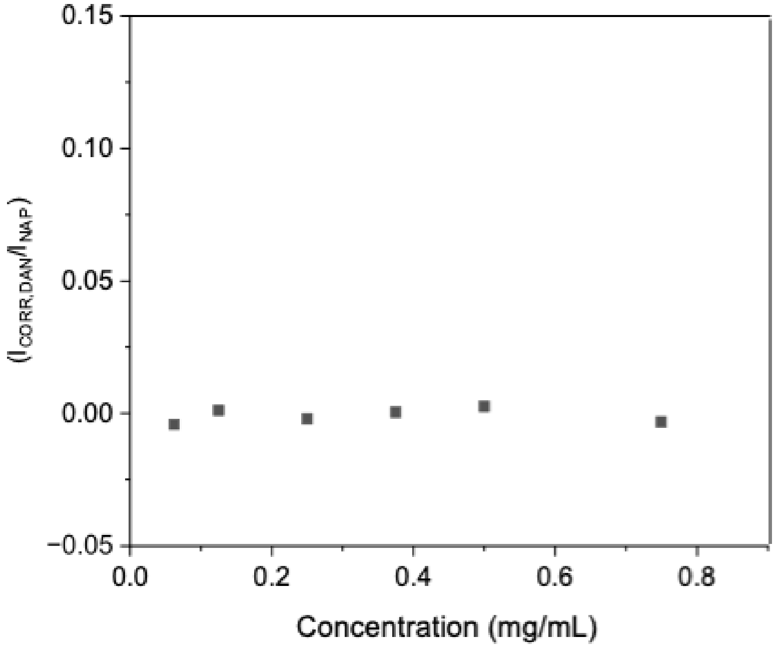

Figure S6. The corrected acceptor-to-donor ratio ($I_{corr,DAN}/I_{NAP}$) obtained by mixing DAN-MEO and NAP-MEO in 1:1 molar ratio at different total peptoid concentrations (0, 0.0625, 0.125, 0.25, 0.375, 0.50, and 0.75 mg/mL) in aqueous solutions). The corrected acceptor-to-donor ratio is calculated using the fluorescent emission data **Figure S5** and Eq. S6. Error bars represent standard deviation from six measurements.

Critical Micellar Concentration (CMC) Measurements

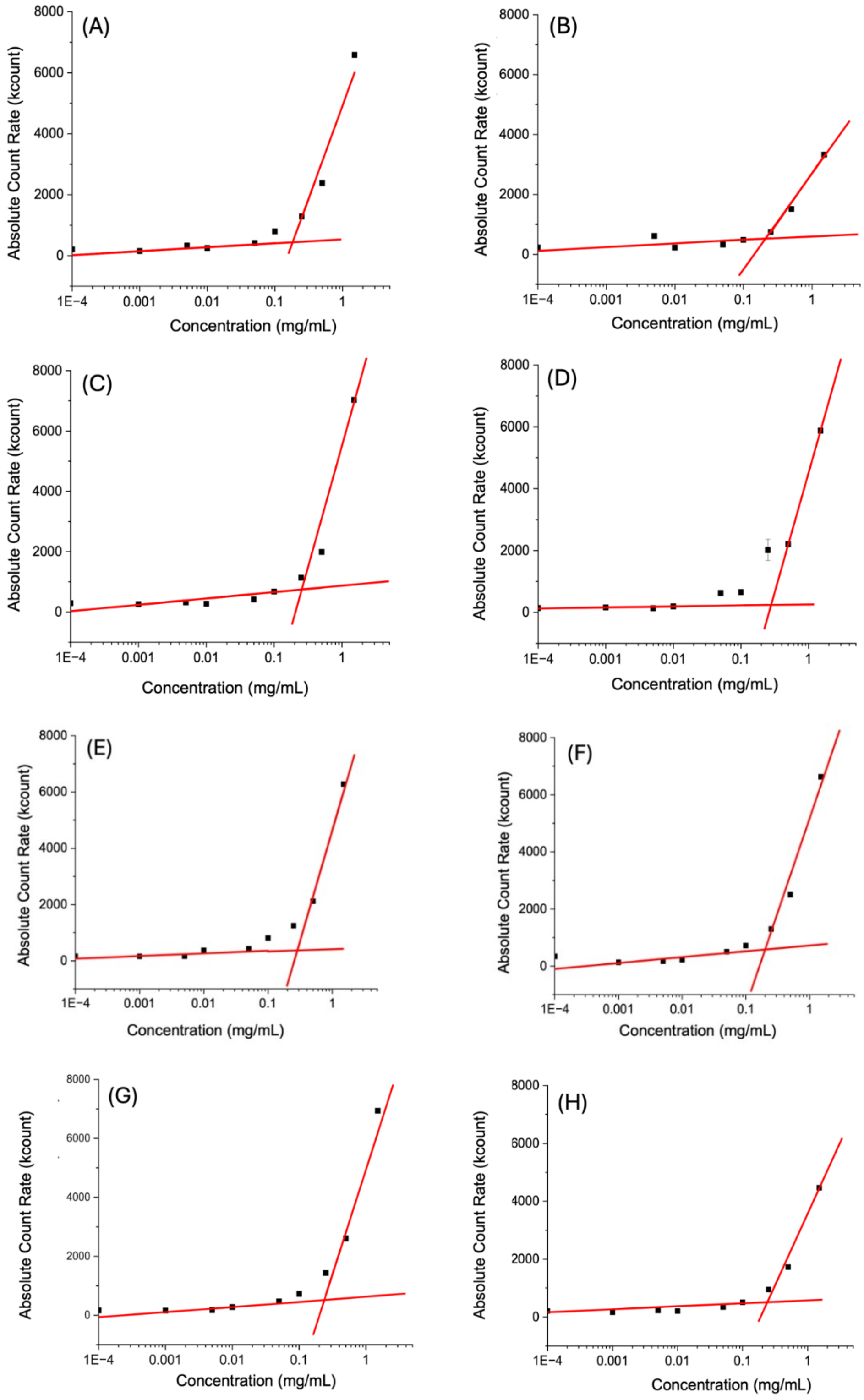

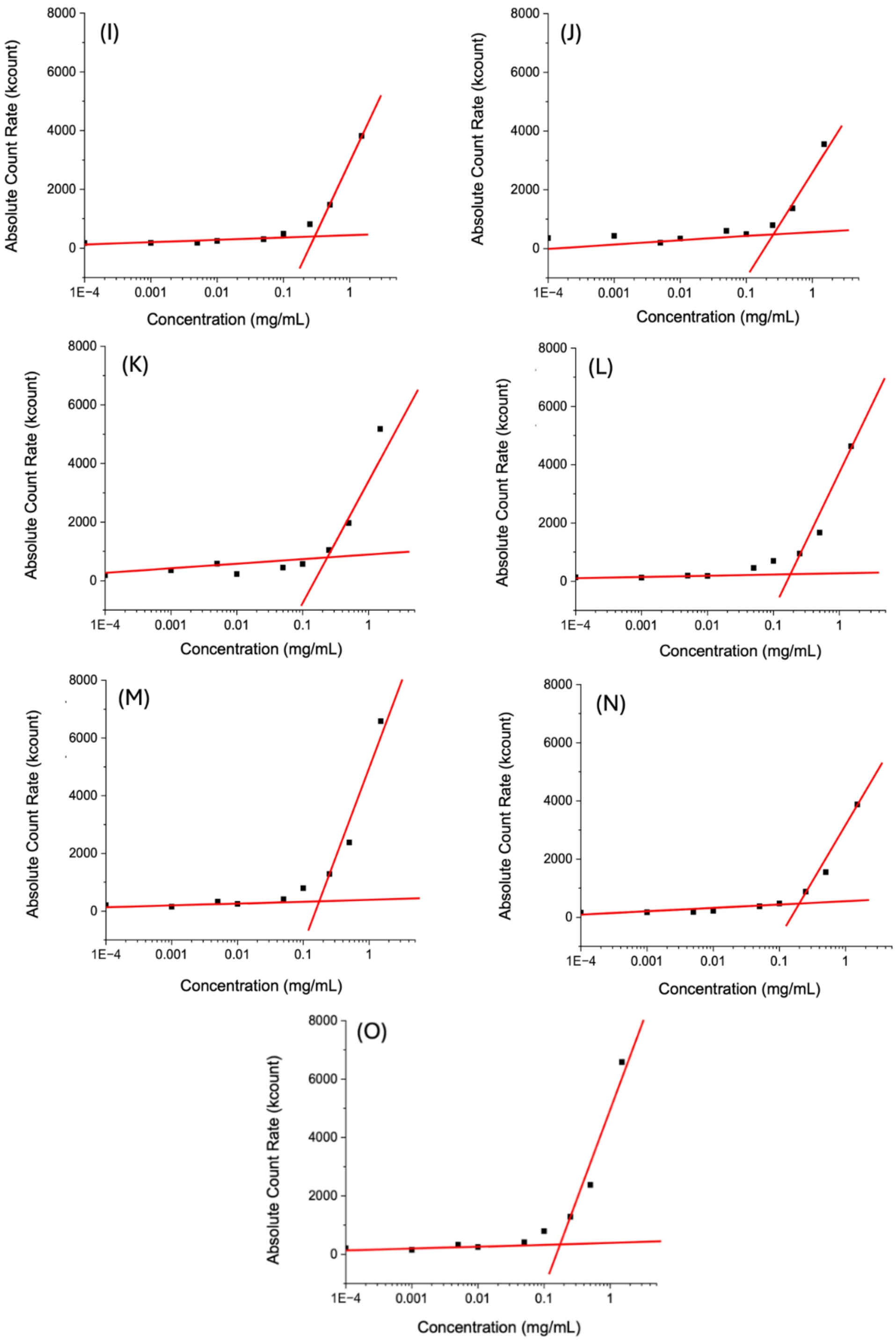

Figure S7. Determination of critical micellar concentration (CMC) by SLS method. Plots of the absolute count rate versus peptoid concentration. (A) SEQ 1, (B) SEQ 2, (C) SEQ 5, (D) SEQ 1: SEQ 5 (2:8), (E) SEQ 1:SEQ 5 (4:6), (F) SEQ 1:SEQ 5 (6:4), (G) SEQ 1:SEQ 5 (8:2), (H) SEQ 2:SEQ 5 (2:8), (I) SEQ 2:SEQ 5 (4:6), (J) SEQ 2:SEQ 5 (6:4), (K) SEQ 2:SEQ 5 (8:2), (L) SEQ 1:SEQ 2 (2:8), **(**M) SEQ 1:SEQ 2 (4:6), (N) SEQ 1:SEQ 2 (6:4), and (O) SEQ 1:SEQ 2 (8:2). The error bars represent standard deviation from triplicate measurements.

Guinier Plots Analysis

According to the Guinier approximation, the intensity $I(Q)$ of the scattered X-rays at small angles (Q < 0.1 Å^{-1}) can be expressed in Eq. S7:

$$I(Q) = I(0)^{-\left(\frac{Q^2R_g^2}{3}\right)} \qquad \text{Eq. S7}$$

where $I(Q)$ represents the scattering intensity as a function of Q, $I(0)$ is the forward scattering intensity at Q = 0, and R_g indicates the radius of gyration of the micellar assemblies. The analysis used the criterion $R_g \times Q < 1.0$ to derive R_g from the plot of ln $I(Q)$ versus Q^2 (**Figure 4B**, **4D**, **4F**). The absolute scattering intensity can be expressed in Eq. S8:

$$I(Q) \approx \phi \Delta\rho^2 v P(Q) \qquad \text{Eq.S8}$$

$I(Q)$ represents the scattering intensity as a function of Q can be applied to the Guinier analysis as shown by, ϕ is the volume fraction for the polymer chain, $\Delta\rho$ is the difference in the scattering length density between the polymer chains and the surrounding H_2O in 60 mM NaCl, and v is the volume of the polymer chains, and $P(Q)$ is the form factor. The Guinier analysis can then be applied to the absolute scattering intensity using the low-q regime, as shown in Eq. S9:

$$P(Q) \approx e^{-1/3Q^2R_g^2} \qquad \text{Eq.S9}$$

Therefore, the aggregation number can be approximated using the $I(0)$ or the forward scattering intensity at Q = 0 from the Guinier analysis (Eq. S10):[S12, S13]

$$I_0 \approx \frac{c}{\rho_m} \Delta\rho^2 v = \frac{c}{\rho_m} \Delta\rho^2 \frac{M_w}{\rho_m N_A} \qquad \text{Eq. S10}$$

Where c is the mass concentration (mass/volume), ρ_m is the mass density of the polymer chain), M_w is the molecular weight of the macromolecule, and N_A is Avogadro's number. If this macromolecule is a micelle in solution, the total molecular mass is the summation of the molecular mass of individual monomers. As a result, $M_w = NM_{m,w}$, where N_{agg} is the aggregation number of a micelle in solution, and $M_{m,w}$ is the molecular mass of the individual polymer chain as described by Eq.S11:[S12, S13]

$$N_{agg} = \frac{M_w}{M_{m,W}} = \frac{I_0}{M_{m,W}} \frac{\rho_m^2 N_A}{c\Delta\rho^2} \qquad \text{Eq.S11}$$

Where N_{agg} is the aggregation number, I_0 is the intensity of $I(Q)$ at $Q \rightarrow 0$, N_A is Avogadro's number, ρ_m is the calculated density of the polymer chain, and ρ_{H_2O} is the scattering length density of H_2O, ρ is the scattering length density of a peptoid chain, $M_{m,w}$ is the molecular weight of the polymer, and c is the concentration of the peptoid solution in 60 mM NaCl. The scattering length densities and the mass densities were calculated from the monomer density of 0.95 g/cm^3, 1.18 g/cm^3, and 0.996 g/cm^3 for *N*-decyl glycine (N_{DE}), *N*-2-methoxyethyl glycine (N_{ME}), and *N*-2-carboxyethyl glycine (N_{CE}), respectively.[S14-S16] The calculated mass densities of SEQ 1 and SEQ 2 from the previously reported monomer densities were 1.079 g/cm^3 and for 1.085 g/cm^3 SEQ 5. The Q-range selected for analysis was chosen such that $R_g \times Q < 1.0$. (Note: The X-ray scattering length densities of the hydrophobic and hydrophilic block of the studied sequence defined peptoid oligomers were determined from the polymer mass densities).

Table S3. Radius of gyration (R_g), aggregation number (N_{agg}), micellar boundary radius (R_m), and CMC of single-sequence micelles and hybrid micelles consisting of two different sequence-defined peptoid BCP in varying molar ratios in aqueous solutions (pH = 9.0, 20 °C).

**Sample Identification**	**R_g (Å)**	**N_{agg}**	**R_m (Å)**	**CMC (mg/mL)**
SEQ 1	54.2 ± 0.5	79.0 ± 0.4	112.02 ± 0.02	0.163 ± 0.002
SEQ 2	43.4 ± 0.4	52.1 ± 0.2	88.26 ± 0.01	0.214 ± 0.005
SEQ 5	56.8 ± 0.5	85.5 ± 0.5	109.11 ± 0.03	0.248 ± 0.002
SEQ 1:SEQ 5 (2:8)	54.6 ±0.6	83.8 ± 0.4	109.65 ± 0.03	0.185 ± 0.002
SEQ 1:SEQ 5 (4:6)	54.1± 0.5	82.7 ± 0.4	110.36 ± 0.03	0.221 ± 0.003
SEQ 1:SEQ 5 (6:4)	53.7± 0.6	82.3 ±0.5	110.73 ± 0.03	0.205± 0.001
SEQ 1:SEQ 5 (8:2)	52.7 ± 0.5	81.1 ± 0.4	111.05 ± 0.03	0.206 ±0.001
SEQ 2:SEQ 5 (2:8)	52.0 ± 0.5	72.9 ± 0.4	103.44 ± 0.02	0.215 ±0.003
SEQ 2:SEQ 5 (4:6)	48.8 ± 0.4	65.5 ± 0.3	100.95 ± 0.02	0.210 ± 0.009
SEQ 2:SEQ 5 (6:4)	46.1 ± 0.5	57.9 ± 0.3	97.7 ± 0.02	0.208 ± 0.009
SEQ 2:SEQ 5 (8:2)	44.3 ± 0.4	53.8 ± 0.3	93.15 ± 0.02	0.234 ± 0.008
SEQ 1:SEQ 2 (2:8)	43.7 ± 0.5	53.0 ± 0.3	94.00 ± 0.03	0.212 ± 0.008
SEQ 1:SEQ 2 (4:6)	46.7 ± 0.6	59.0 ± 0.3	98.4 ± 0.03	0.223 ± 0.004
SEQ 1:SEQ 2 (6:4)	48.1 ± 0.7	64.0 ± 0.3	101.66 ± 0.02	0.228 ±0.004
SEQ 1: SEQ 2 (8:2)	52.1± 0.7	73.6 ± 0.5	104.00± 0.02	0.210 ± 0.005

Table S4. Fitting parameters for radius of gyration (R_g) including fitting q-range, $q \times R_g$ fitting range, and R^2.

Sample Identification	q-Range	$q \times R_g$ Range	R^2
SEQ 1	$0.001052 < q < 0.01856$	$0.5717 < q \times R_g < 1.0085$	0.9884
SEQ 2	$0.01029 < q < 0.02306$	$0.4462 < q \times R_g < 1.0001$	0.9728
SEQ 5	$0.00934 < q < 0.01762$	$0.5308 < q \times R_g < 1.0011$	0.9832
SEQ 1:SEQ 5 (2:8)	$0.00887 < q < 0.01833$	$0.5684 < q \times R_g < 1.0007$	0.9813
SEQ 1:SEQ 5 (4:6)	$0.00934 < q < 0.01856$	$0.5053 < q \times R_g < 1.0042$	0.9835
SEQ 1:SEQ 5 (6:4)	$0.01076 < q < 0.1856$	$0.4462 < q \times R_g < 0.9967$	0.9727
SEQ 1:SEQ 5 (8:2)	$0.01029 < q < 0.01904$	$0.5425 < q \times R_g < 1.0039$	0.9824
SEQ 2:SEQ 5 (2:8)	$0.0005 < q < 0.01927$	$0.5229 < q \times R_g < 1.0027$	0.9852
SEQ 2:SEQ 5 (4:6)	$0.00887 < q < 0.02045$	$0.433 < q \times R_g < 0.9984$	0.9854
SEQ 2:SEQ 5 (6:4)	$0.00934 < q < 0.02187$	$0.4305 < q \times R_g < 1.0082$	0.9777
SEQ 2:SEQ 5 (8:2)	$0.01052 < q < 0.02258$	$0.4667 < q \times R_g < 1.0015$	0.9739
SEQ 1:SEQ 2 (2:8)	$0.00981 < q < 0.02282$	$0.429 < q \times R_g < 0.9975$	0.9742
SEQ 1:SEQ 2 (4:6)	$0.0091 < q < 0.02164$	$0.425 < q \times R_g < 1.01$	0.9704
SEQ 1:SEQ 2 (6:4)	$0.1147 < q < 0.02093$	$0.5515 < q \times R_g < 1.0064$	0.9716
SEQ 1: SEQ 2 (8:2)	$0.011 < q < 0.01951$	$0.5733 < q \times R_g < 1.017$	0.9652

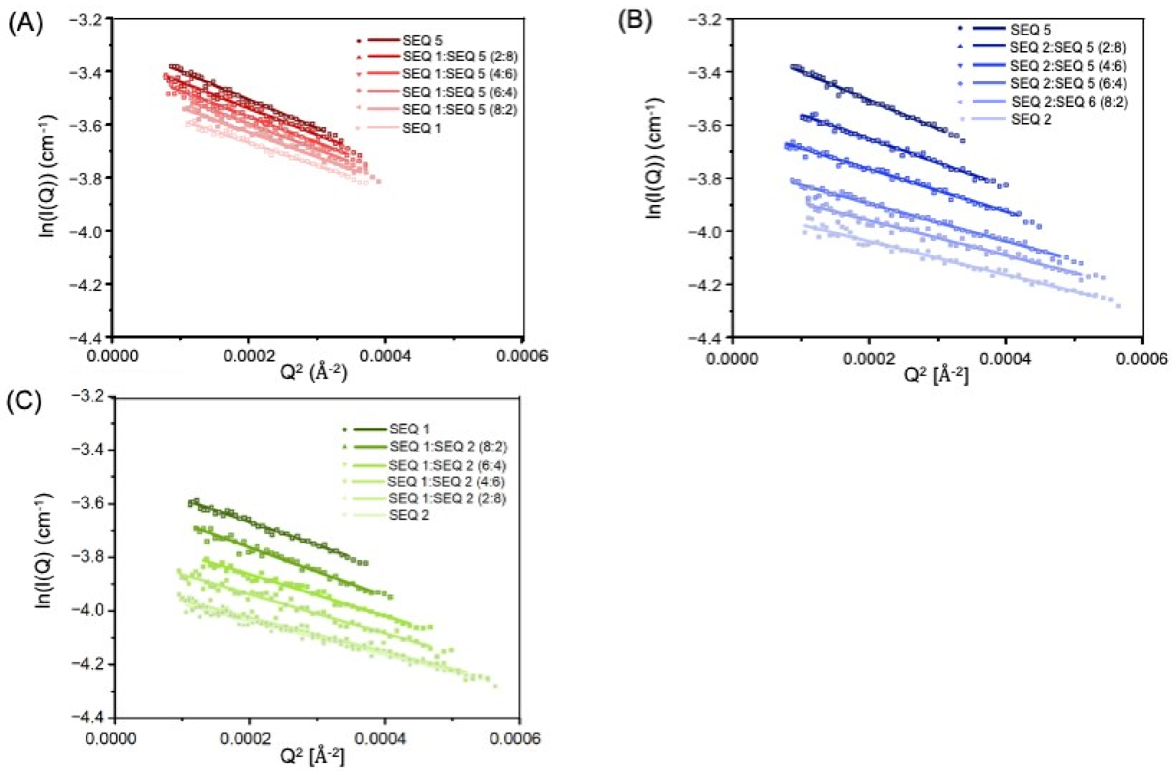

Figure S8. The corresponding ln[$I(Q)\times A$] vs. Q^2 plots: (A) SEQ 1: SEQ 5; (B) SEQ 2: SEQ 5; (C) SEQ 2: SEQ 1 to the SAXS profiles of hybrid micelles consisting of two different sequence-defined peptoid BCPs in varying molar ratios ([peptoid]= 3.0 mg/mL, [NaCl]=60 mM, pH = 9.0, 20 °C) in **Figure 4**.

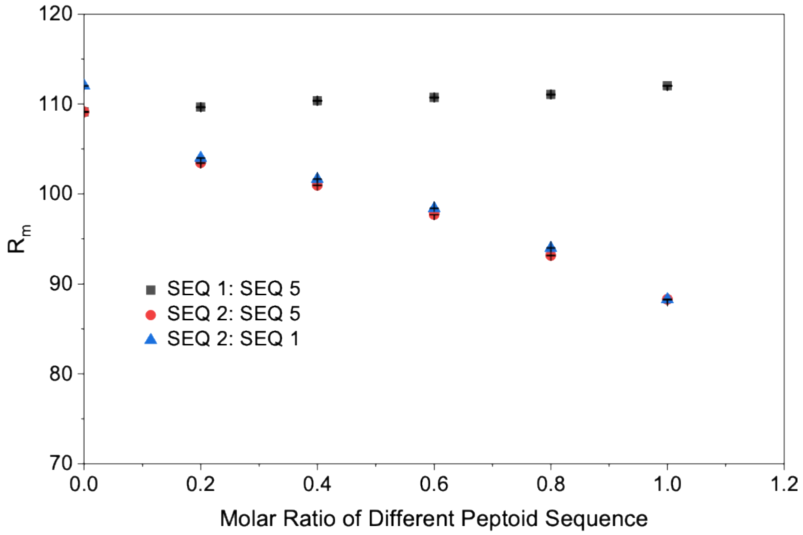

Figure S9. The micellar radius (R_m, Å) for each hybrid micelle at varying molar ratios of two peptoid sequences (SEQ 1: SEQ 5, SEQ 2: SEQ 5, SEQ 2: SEQ 1).

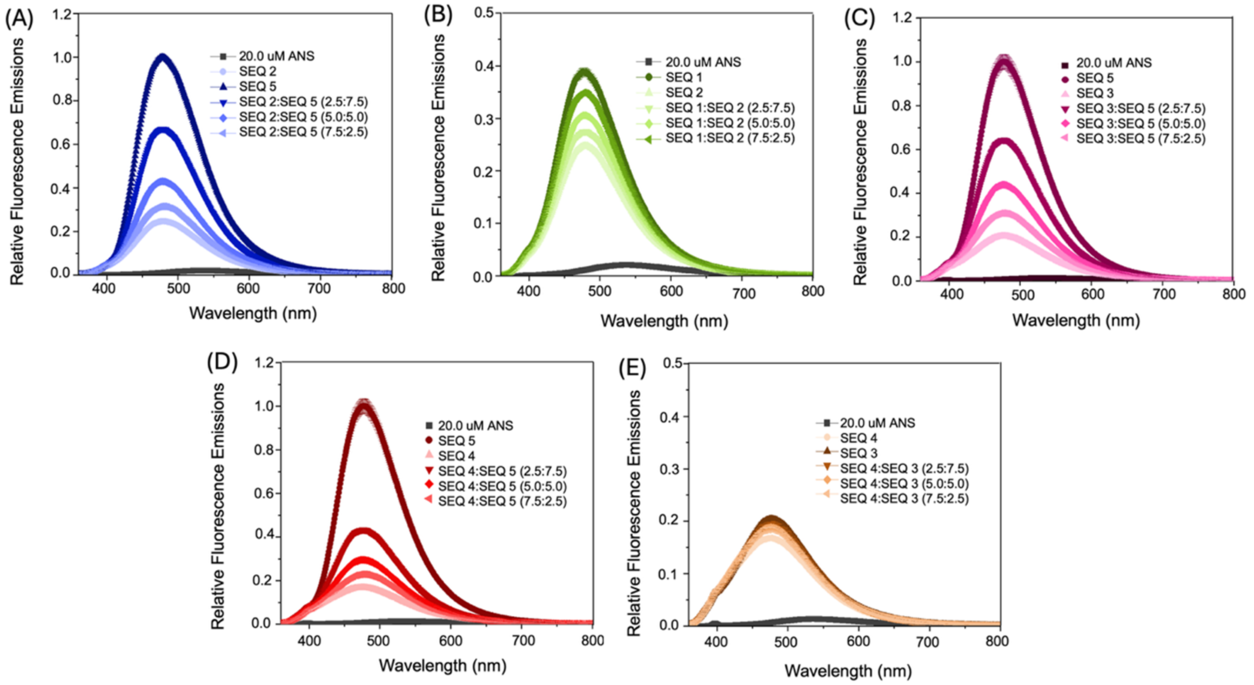

Figure S10. Relative fluorescence emission spectra of aqueous solutions of single-sequence micelles or hybrid micelles consisting of two different peptoid sequences (A) SEQ 2 and SEQ 5, (B) SEQ 1 and SEQ 2, (C) SEQ 3 and SEQ 5, (D) SEQ 4 and SEQ 5, and (E) SEQ 3 and SEQ 4 in varying molar ratios and ANS fluorescence probe (20 μM). The relative fluorescence intensity was calculated by dividing the fluorescence emissions intensity of the sample of interest by the corresponding fluorescence emissions intensity of SEQ 5. The error bars represent standard deviation from six measurements.

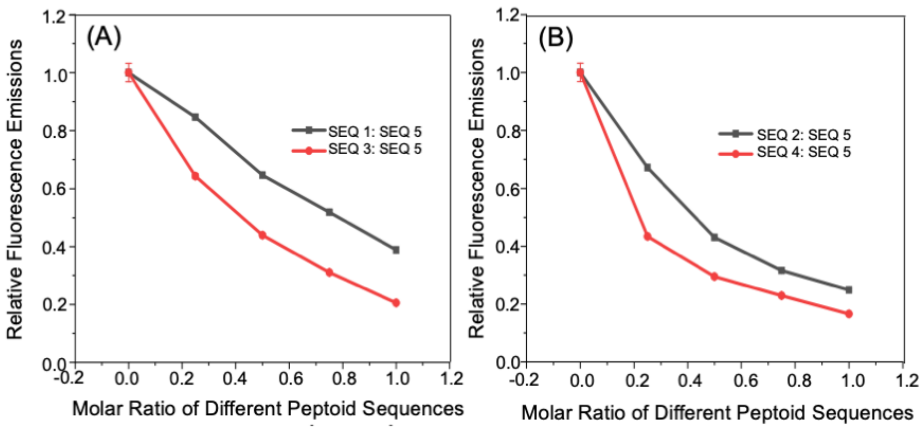

Figure S11. Plots of relative maximum fluorescence intensity of hybrid micelles at different molar ratios of the two different peptoid sequences: (A) SEQ 1: SEQ 5 and SEQ 3: SEQ 5; (B) SEQ 2: SEQ 5 and SEQ 4: SEQ 5. The spectra show an intensity maximum at 480-500 nm. Error bars are standard deviation from triplicate measurements.

Table S5. The average maximum relative fluorescence emissions of aqueous solutions of single-sequence micelles or hybrid micelles consisting of two different peptoid sequences (A) SEQ 2 and SEQ 5, (B) SEQ 1 and SEQ 2, (C) SEQ 3 and SEQ 5, (D) SEQ 4 and SEQ 5, and (E) SEQ 3 and SEQ 4 in varying molar ratios and ANS fluorescence probe (20 μM). The relative fluorescence intensity was calculated by dividing the fluorescence emissions intensity of the sample of interest by the corresponding fluorescence emissions intensity of SEQ 5. The error bars represent standard deviation from six measurements.

Sample Name	Relative Maximum Fluorescence Intensity
SEQ 1	0.388 ± 0.008
SEQ 2	0.248 ± 0.005
SEQ 3	0.204 ± 0.003
SEQ 4	0.166 ± 0.002
SEQ 5	1.000 ± 0.031
SEQ 1:SEQ 5 (2.5:7.5)	0.846 ± 0.008
SEQ 1:SEQ 5 (5.0:5.0)	0.646 ± 0.007
SEQ 1:SEQ 5 (7.5:2.5)	0.519 ± 0.007
SEQ 2:SEQ 5 (2.5:7.5)	0.672 ± 0.002
SEQ 2:SEQ 5 (5.0:5.0)	0.431 ± 0.003
SEQ 2:SEQ 5 (7.5:2.5)	0.316 ± 0.002
SEQ 1:SEQ 2 (2.5:7.5)	0.275 ± 0.003
SEQ 1:SEQ 2 (5.0:5.0)	0.306 ± 0.003
SEQ 1:SEQ 2 (7.5:2.5)	0.349 ± 0.001
SEQ 3:SEQ 5 (2.5:7.5)	0.643 ± 0.007
SEQ 3:SEQ 5 (5.0:5.0)	0.440 ± 0.007
SEQ 3:SEQ 5 (7.5: 2.5)	0.310 ± 0.003
SEQ 4:SEQ 5 (2.5:7.5)	0.434 ± 0.004
SEQ 4:SEQ 5 (5.0:5.0)	0.295 ± 0.005
SEQ 4:SEQ 5 (7.5:2.5)	0.229 ± 0.002
SEQ 3:SEQ 4 (2.5:7.5)	0.186 ± 0.005
SEQ 3:SEQ 4 (5.0:5.0)	0.189 ± 0.002
SEQ 3:SEQ 4 (7.5:2.5)	0.194 ± 0.004

References

(S1) Zuckermann, R. N.; Kerr, J. M.; Kent, S. B. H.; Moos, W. H. Efficient method for the preparation of peptoids [oligo(N-substituted glycines)] by submonomer solid-phase synthesis. *Journal of the American Chemical Society* **1992**, *114* (26), 10646-10647. DOI: 10.1021/ja00052a076.

(S2) Sternhagen, G. L.; Gupta, S.; Zhang, Y.; John, V.; Schneider, G. J.; Zhang, D. Solution self-assemblies of sequence-defined ionic peptoid block copolymers. *Journal of the American Chemical Society* **2018**, *140* (11), 4100-4109.

(S3) Topel, Ö.; Çakır, B. A.; Budama, L.; Hoda, N. Determination of critical micelle concentration of polybutadiene-block-poly(ethyleneoxide) diblock copolymer by fluorescence spectroscopy and dynamic light scattering. *Journal of Molecular Liquids* **2013**, *177*, 40-43. DOI: 10.1016/j.molliq.2012.10.013.

(S4) Kroll, P.; Benke, J.; Enders, S.; Brandenbusch, C.; Sadowski, G. Influence of Temperature and Concentration on the Self-Assembly of Nonionic CiEj Surfactants: A Light Scattering Study. *ACS Omega* **2022**, *7* (8), 7057-7065. DOI: 10.1021/acsomega.1c06766.

(S5) Khougaz, K.; Gao, Z.; Eisenberg, A. Determination of the Critical Micelle Concentration of Block Copolymer Micelles by Static Light Scattering. *Macromolecules* **1994**, *27* (22), 6341-6346. DOI: 10.1021/ma00100a017.

(S6) Hopkins, J. BioXTAS RAW 2: new developments for a free open-source program for small-angle scattering data reduction and analysis. *Journal of Applied Crystallography* **2024**, *57* (1), 194-208. DOI:10.1107/S1600576723011019.

(S7) Doucet, M. SasView version 5.0. **2019**. DOI: 10.5281/zenodo.3011184.

(S8) Hansen, C. Hansen Solubility Parameters, 2ed.; CRC Press, **2007**. DOI: 10.1201/9781420006834.

(S9) Van Krevelen, D. W.; Te Nijenhuis, K. Properties of polymers: their correlation with chemical structure; their numerical estimation and prediction from additive group contributions; Elsevier, **2009**.

(S10) Gong, W. Design of Active Pharmaceutical Ingredients Solid States in Crystallization Processes. The University of Western Ontario, Electronic Thesis and Dissertation Repository, **2024**.

(S11) Imai, S.; Takenaka, M.; Sawamoto, M.; Terashima, T. Self-Sorting of Amphiphilic Copolymers for Self-Assembled Materials in Water: Polymers Can Recognize Themselves. *Journal of the American Chemical Society* **2019**, *141* (1), 511-519. DOI: 10.1021/jacs.8b11364.

(S12) Nayem, J.; Zhang, Z.; Tomlinson, A.; Zarraga, I. E.; Wagner, N. J.; Liu, Y. Micellar Morphology of Polysorbate 20 and 80 and Their Ester Fractions in Solution via Small-Angle Neutron Scattering. *J Pharm Sci* **2020**, *109* (4), 1498-1508. DOI: 10.1016/j.xphs.2019.12.016.

(S13) Liu, Y.; Chen, S.-H.; Huang, J. S. Small-Angle Neutron Scattering Analysis of the Structure and Interaction of Triblock Copolymer Micelles in Aqueous Solution. *Macromolecules* **1998**, *31* (7), 2236-2244. DOI: 10.1021/ma971253o.

(S14) Murnen, H. K.; Rosales, A. M.; Dobrynin, A. V.; Zuckermann, R. N.; Segalman, R. A. Persistence length of polyelectrolytes with precisely located charges. *Soft Matter* **2013**, *9* (1), 90-98, 10.1039/C2SM26849C. DOI: 10.1039/C2SM26849C.

(S15) Rosales, A. M.; McCulloch, B. L.; Zuckermann, R. N.; Segalman, R. A. Tunable Phase Behavior of Polystyrene–Polypeptoid Block Copolymers. *Macromolecules* **2012**, *45* (15), 6027-6035. DOI: 10.1021/ma300625b.

(S16) Sun, J.; Jiang, X.; Lund, R.; Downing, K. H.; Balsara, N. P.; Zuckermann, R. N. Self-assembly of crystalline nanotubes from monodisperse amphiphilic diblock copolypeptoid tiles. *Proc Natl Acad Sci U S A* **2016**, *113* (15), 3954-3959. DOI: 10.1073/pnas.1517169113.
